# Integrated Model of *De Novo* and Inherited Genetic Variants Yields Greater Power to Identify Risk Genes

**DOI:** 10.1371/journal.pgen.1003671

**Published:** 2013-08-15

**Authors:** Xin He, Stephan J. Sanders, Li Liu, Silvia De Rubeis, Elaine T. Lim, James S. Sutcliffe, Gerard D. Schellenberg, Richard A. Gibbs, Mark J. Daly, Joseph D. Buxbaum, Matthew W. State, Bernie Devlin, Kathryn Roeder

**Affiliations:** 1 Lane Center of Computational Biology, Carnegie Mellon University, Pittsburgh, Pennsylvania, United States of America; 2 Departments of Psychiatry and Genetics, Yale University School of Medicine, New Haven, Connecticut, United States of America; 3 Department of Statistics, Carnegie Mellon University, Pittsburgh, Pennsylvania, United States of America; 4 Seaver Autism Center for Research and Treatment, Icahn Mount Sinai School of Medicine, New York, New York, United States of America; 5 Department of Psychiatry, Icahn Mount Sinai School of Medicine, New York, New York, United States of America; 6 Analytic and Translational Genetics Unit, Department of Medicine, Massachusetts General Hospital and Harvard Medical School, Boston, Massachusetts, United States of America; 7 Program in Medical and Population Genetics, Broad Institute of Harvard and MIT, Cambridge, Massachusetts, United States of America; 8 Vanderbilt Brain Institute, Departments of Molecular Physiology & Biophysics and Psychiatry, Vanderbilt University, Nashville, Tennessee, United States of America; 9 Pathology and Laboratory Medicine, Perelman School of Medicine, University of Pennsylvania, Philadelphia, Pennsylvania, United States of America; 10 Human Genome Sequencing Center, Baylor College of Medicine, Houston, Texas, United States of America; 11 Department of Genetics and Genomic Sciences, Icahn Mount Sinai School of Medicine, New York, New York, United States of America; 12 Friedman Brain Institute, Icahn Mount Sinai School of Medicine, New York, New York, United States of America; 13 Department of Psychiatry, University of Pittsburgh School of Medicine, Pittsburgh, Pennsylvania, United States of America; Dartmouth College, United States of America

## Abstract

*De novo* mutations affect risk for many diseases and disorders, especially those with early-onset. An example is autism spectrum disorders (ASD). Four recent whole-exome sequencing (WES) studies of ASD families revealed a handful of novel risk genes, based on independent *de novo* loss-of-function (LoF) mutations falling in the same gene, and found that *de novo* LoF mutations occurred at a twofold higher rate than expected by chance. However successful these studies were, they used only a small fraction of the data, excluding other types of *de novo* mutations and inherited rare variants. Moreover, such analyses cannot readily incorporate data from case-control studies. An important research challenge in gene discovery, therefore, is to develop statistical methods that accommodate a broader class of rare variation. We develop methods that can incorporate WES data regarding *de novo* mutations, inherited variants present, and variants identified within cases and controls. TADA, for Transmission And *De novo* Association, integrates these data by a gene-based likelihood model involving parameters for allele frequencies and gene-specific penetrances. Inference is based on a Hierarchical Bayes strategy that borrows information across all genes to infer parameters that would be difficult to estimate for individual genes. In addition to theoretical development we validated TADA using realistic simulations mimicking rare, large-effect mutations affecting risk for ASD and show it has dramatically better power than other common methods of analysis. Thus TADA's integration of various kinds of WES data can be a highly effective means of identifying novel risk genes. Indeed, application of TADA to WES data from subjects with ASD and their families, as well as from a study of ASD subjects and controls, revealed several novel and promising ASD candidate genes with strong statistical support.

## Author Summary

The genetic underpinnings of autism spectrum disorder (ASD) have proven difficult to determine, despite a wealth of evidence for genetic causes and ongoing effort to identify genes. Recently investigators sequenced the coding regions of the genomes from ASD children along with their unaffected parents (ASD trios) and identified numerous new candidate genes by pinpointing spontaneously occurring (*de novo*) mutations in the affected offspring. A gene with a severe (*de novo*) mutation observed in more than one individual is immediately implicated in ASD; however, the majority of severe mutations are observed only once per gene. These genes create a short list of candidates, and our results suggest about 50% are true risk genes. To strengthen our inferences, we develop a novel statistical method (TADA) that utilizes inherited variation transmitted to affected offspring in conjunction with (*de novo*) mutations to identify risk genes. Through simulations we show that TADA dramatically increases power. We apply this approach to nearly 1000 ASD trios and 2000 subjects from a case-control study and identify several promising genes. Through simulations and application we show that TADA's integration of sequencing data can be a highly effective means of identifying risk genes.

## Introduction

The genetic architecture of autism spectrum disorders (ASD) is complex and thought to involve the action of at least hundreds of genes. Yet, despite this complexity, four recent studies [1]–[4] identified five novel genes affecting the risk for ASD from whole-exome sequencing (WES) of 932 ASD probands. The studies made these discoveries by also sequencing the parents of the probands and thereby discovering a multiplicity of independent Loss-of-Function (LoF) mutations in each of these five genes. The multiplicity is key: due to the rarity of *de novo* LoF events, two or more independent recurrent events in a sample of this size generate more evidence for association than would two LoF variants found in a comparable case and control sample. Thus, even though *de novo* events are rare, these observations provide an excellent signal-to-noise ratio, have proven valuable in the pursuit of reliable signals for genes affecting the ASD risk, and are likely to form the foundation for many studies targeting gene discovery in the future [5].

Note, however, that the multiplicity test is using only a small fraction of all the information collected by a WES study. Many other *de novo* events occur, beyond LoF, and these are ignored. Moreover it completely ignores inherited rare variants within families. And, of course, delineation of rare variants into inherited and *de novo* is challenging or impossible for case-control studies. We conjecture that the distribution of variation, whether inherited, *de novo* and from case-control, can be leveraged, in combination with the *de novo* mutations, to maximize the statistical power to detect risk genes.

We propose an integrated model of *de novo* mutations and transmitted variation to address these challenges. We demonstrate that both the number of *de novo* mutations and the numbers of different types of transmitted variations in family trios (father, mother and an affected child), follow simple distributions dependent on a set of common parameters: mutation rates, relative risks of mutations and population frequency of the variants. This model readily incorporates additional data from case-control studies. The statistical framework of our model enables us to rigorously analyze the genetic architecture of a complex disease, conduct power and sample size analysis, and identify risk genes with higher sensitivity. Through simulations we show that the power of our novel statistical test, called TADA for “transmission and *de novo* association”, is substantially higher than competing tests. Our simulations also provide guidance in planning future studies targeting discovery of genes involved in the risks of complex diseases, henceforth, risk genes.

We demonstrate the benefits of TADA through an extensive study of ASD using published WES data from 932 ASD trios as well as nearly 1000 ASD subjects and matched control subjects from the ARRA Autism Sequencing Consortium (AASC) study [6], [7]. Using the model underlying TADA, we estimate there are approximately 1000 genes that play a role in risk for ASD, with an average relative risk of approximately 20 due to LoF in one of these genes. Finally, we identify several potential novel ASD risk genes (genes whose mutations affect the risk of ASD) using TADA and the ASD data.

## Results

### Multiplicity test of *de novo* mutations

For concreteness we start by reviewing the *multiplicity test* to detect risk genes by evaluating the independent recurrence of *de novo* mutations in the same gene. The multiplicity test classifies a gene as affecting risk if it sustains 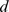 or more recurrent *de novo* LoF mutations in a sample of 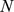 families. Based on computations of expected rates of *de novo* events as a function of a gene's exonic length and base pair composition [2], a recent study [1] found that 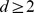 LoF events for 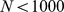 is significant evidence to declare a gene as a risk gene (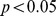, genomewide). Applying this threshold to data from four ASD family studies [1]–[4] led to the discovery of five novel genes affecting ASD risk.

A weakness of the multiplicity test is that it produces a single threshold for the entire genome, regardless of the heterogeneity amongst genes in their sizes and base pair composition, and its threshold is a function of sample size, so that the threshold for 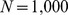 is inadequate when the sample increases to 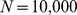. To illustrate the power of the Multiplicity Test and its properties, we performed some simulations using genetic parameters that are described and estimated in the next section.

**Figure 1:**
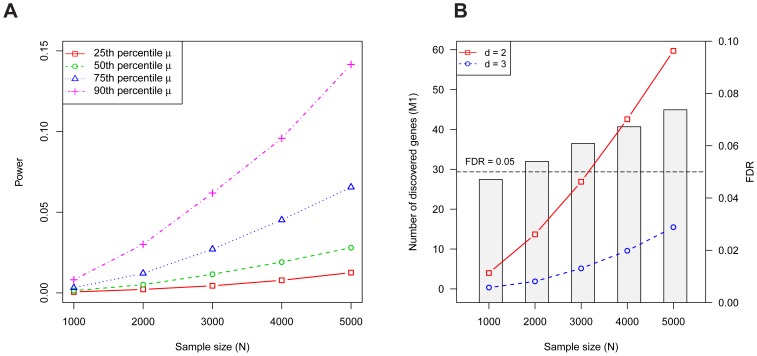
Properties of the Multiplicity Test. (A) The probability a risk gene has two or more *de novo* LoF mutations in 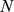 families (i.e., the power) depends on the mutation rate 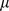. Power per gene of the Multiplicity Test as a function of 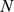 is shown for 4 mutation rates, which were chosen based on percentiles (25'th, 50'th, 75'th, 90'th) of the distribution of 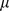 obtained from the full gene set. (B) The expected number of risk genes discovered by the Multiplicity Test at 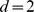 (red, solid) or 3 (blue, dashed) as a function of the sample size 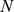. The barplot shows the FDR at 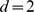. The simulation assumes 1000 diseases genes out of 18,000, each with relative risk 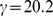; these parameters were estimated in the section on Genetic Architecture of ASD.

As demonstrated previously [1], the power for detecting a gene increases monotonically with increasing sample size 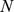 and it depends strongly on the gene's mutation rate (Figure 1A). Although the per gene power is relatively low, for a disorder like ASD, more than 60 genes are expected to contain at least two LoF mutations with 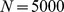 families (Figure 1B). The corresponding false discovery rate (FDR) is less than 5% for 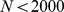 and well below 10% for 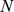 as large as 5,000; switching to a threshold of 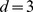 to diminish false discoveries leads to a significant loss in power (Figure 1B).

The original treatment of the multiplicity test as requiring a single threshold is simple to adjust. Instead one can compute the *p*-value for each gene using a Poisson model for the probability of observing 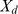 or more recurrent *de novo* events based on the gene's mutation rate. We will call such a test the *De Novo Test*. This test automatically incorporates the number of families and a gene specific mutation rate to determine the likelihood of recurrent *de novo* events.

### Model of *de novo* and inherited mutations in a family design

TADA model is formulated for sequence data from individual genes. Data for the model can come from sequences of trios (unaffected parents and an affected child) and from cases and controls. Given the information from a gene, namely the pattern of *de novo* mutations and inherited, damaging variants in the affected progeny, the goal is to relate the data with the underlying genetic parameters such as the relative risk of the mutations. In the model, we restrict the class of variation to rare and deleterious mutations acting dominantly and assume subjects can be classified as carrying one of two “alleles”, those with a deleterious mutation of this type (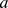) and those without (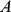). We put alleles in quotes because, for example, we treat all LoF events in the same gene as a single LoF “allele”. Because severe mutations are generally present at very low frequencies in the population (typically 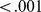), there are effectively two possible genotypes per gene, 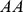 and 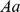. If we let 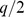 denote the allele frequency of 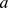, then the frequencies of the genotypes 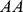 and 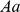 in the population are approximately 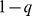 and 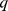, respectively.

**Figure 2:**
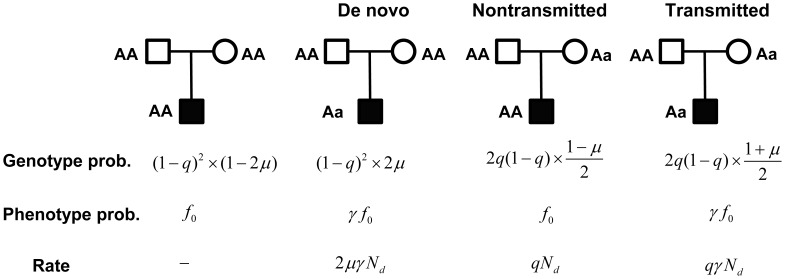
A probabilistic model for a family trio with an affected child. Genotype probabilities are computed as the marginal probability of parental genotypes times the conditional probability of the child, given the parents. The parameters 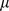 and 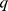 represent the mutation rate, and the population frequency of the 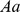 genotype, respectively. Phenotype probabilities for the child, given genotype, are a function of 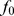, the penetrance of the 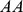 genotype, and 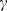 the relative risk of the mutation 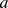. Rate is the (approximate) rate of observing counts 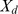, 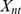 and 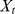 from the latter 3 types of trios, respectively.

For a trio consisting of unaffected parents and an affected child, there are four likely genotype combinations (Figure 2), of which only three are informative: if both parents are homozygous, a heterozygous child results from a *de novo* mutation; and if one parent is heterozygous, the 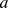 allele is either transmitted or not. Based on the *de novo* and transmitted alleles, we formulate a likelihood model for the observed data. Let 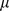 denote the rate of mutation for the gene being analyzed per generation and chromosome; let 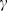 denote the genotype relative risk for the genotype 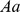; and let 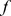 and 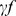 denote the penetrance of 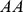 and 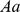, respectively. Let 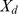, 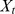 and 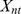 be the counts of each of the three outcomes (*de novo*, transmitted and nontransmitted, respectively), from a sample consisting of 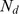 families. These counts approximately follow Poisson distributions (see Text S1 for derivation): 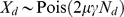, 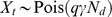, and 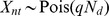.

For case-control data, counts of genotype 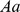 in 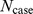 cases and 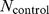 controls follow a Poisson distribution with approximate rate parameters 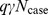 and 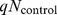, respectively (see Text S1). From this structure it is apparent that the transmitted counts can be viewed as a type of case-control data with sample size 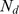. Combining data, let 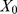 be the total number of 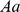 in the controls plus the number of transmitted 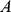 variants, and let 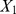 be the total number of 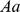 in the cases plus the number of transmitted 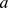 variants. It follows that
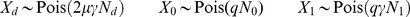
(1)for which 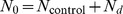 and 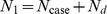. The resulting probability model has three parameters (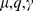) per gene. For each gene, the mutation rate per gene (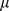) can be estimated from its exonic length and nucleotide content [1] and hence this quantity can be treated as known. The statistical problem for each gene is to estimate 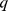 and then test if 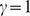.

### Transmission And *De novo* Association test: TADA

We conjecture that a more powerful strategy to discover risk genes from family data is to combine the information on *de novo* and inherited mutations into an unified statistical framework, such as the one we just proposed, which forms the basis for TADA. TADA tests the hypothesis 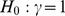 against the alternative 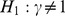. A traditional likelihood ratio test will not work well in this setting because one or more of the counts will be zero for many genes, leading to poor maximum likelihood estimates for 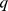 and 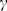. To circumvent this problem we cast TADA in a Hierarchical Bayes (HB) framework, thereby improving estimates of 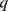 and 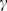 by pooling information across all genes, but still modeling rates as gene-specific. The underlying assumption is that LoF and severe missense mutations are rare in all genes and hence we can learn about the frequency distribution in a given gene by looking at the distribution across all genes. Likewise, we can learn about how mutations in one gene affect risk by examining the range and distribution of risks across all disease-related genes.

The HB model assumes a fraction 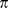 of the genes are associated with the disorder (model 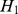); the remaining fraction follow the null model (model 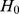). Under 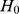, the relative risk is constrained (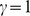), but under 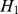, 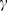 is assumed to follow a distribution across risk genes. For both models, the frequency of severe mutations per gene, 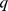, is assumed to vary by gene, with some commonality across the genome. The distributions of 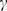 and 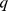 under both models are specified by prior parameters, and we estimate the values of these parameters by maximizing the marginal likelihood of the data (this is known as the Empirical Bayes method, see Methods). Once the prior parameters are estimated, we compute the evidence for 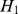 and 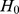 for each gene. Specifically, for the *i*-th gene, let 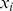 be its data, the evidence for 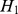 is defined as:
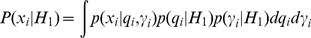
(2)where 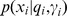 is given by Equation 1, 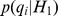 and 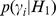 represent the prior distributions. Unlike the likelihood-based test, the evidence for 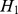 is not based on point estimates of 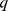 and 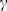; instead it integrates out the two parameters. The model evidence of 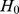 can be defined similarly, except that 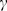 is fixed at 1. The Bayes factor of any gene is the ratio of 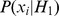 to 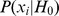. The statistical significance of the Bayes factor is given by its *p*-value, determined empirically by simulating data under the model assuming 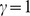 (see Text S1).

Some insights into the relationship to a likelihood-ratio test (LRT) can be gained by examining an approximation of 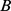, the Bayes factor:
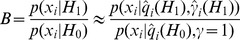
(3)where the parameters are estimated by Bayesian mean posterior estimators. These parameter estimates are a weighted average of the maximum-likelihood estimate for the i-th gene and the mean of the prior distributions. For example, 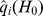 is interpolated between the allele frequency derived from all genes and the gene-specific estimate (Figure S1). Thus the Bayes factor is similar to the LRT except that we utilize a refined estimator of the allele frequency.

The model just described is designed for a single type of mutation (say LoF), but it can incorporate multiple types. For different types of mutations, such as LoF and damaging missense mutations, the distributions of 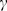 and 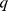 are likely to be different, so we model each type of mutation and estimate the prior parameters separately using the HB framework. Then the total Bayes factor of a gene is the product of the Bayes factor from each type of mutation, and the *p*-value can be computed similarly from simulations. In practice, we note that the damaging missense mutations predicted by bioinformatic tools likely contain a number of mutations having no effect on the gene function, thus we introduce an additional model to account for this feature, downweighting the evidence from missense mutations (see Methods).

The TADA method we described can also be used for *de novo* data alone. Basically, we ignore inherited and standing variants, but allow multiple types of *de novo* mutations. The details are not repeated here, but are provided in our supporting Website (see Methods). We call this simplified model, TADA-Denovo, and it is particularly useful for genes with multiple *de novo* events in different categories (e.g. some nonsense and some missense mutations).

### Genetic architecture of ASD

We use the proposed model to estimate the number of ASD risk genes (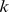), their average relative risk (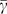), and the distribution of the population frequency of the mutations. These estimates yield insight into the genetics of ASD and pave the way for realistic simulations to study the power of statistical tests. Our overall strategy is first to use *de novo* mutations to estimate an approximate range of the parameter values, then use the HB method to refine these estimates using both family and the case-control data.

**Figure 3:**
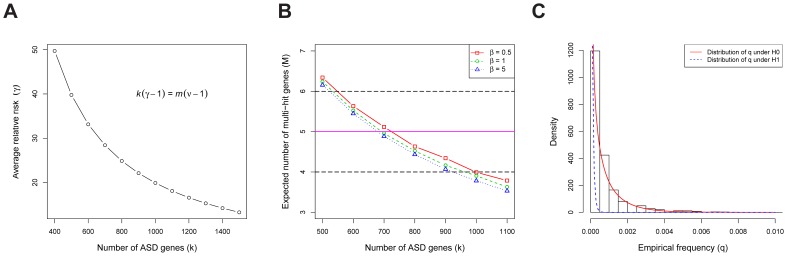
The genetic parameters of ASD. (A) The relationship between the number of ASD risk genes (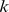) and the average relative risk (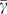). 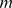 stands for the total number of genes in the human genome, and 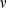 for the fold enrichment of the *de novo* LoF mutations in probands vs. siblings (about 2 in our data). (B) The expected number of multi-hit genes (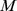) in 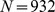 families, as a function of the number of ASD risk genes (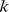). The observed 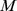 is 5, and we define the plausible range of 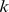 as the values corresponding to 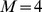 to 6. The model assumes the relative risks of ASD risk genes follow a gamma distribution with the scale parameter 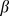. The variance of the relative risk (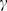) across genes equals 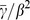 (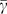 is the average of 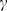 of all ASD risk genes), which limits the range of plausible values for the model. The estimated value of the average 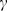 is approximately 20. (C) For each gene, we compute the empirical allele frequency (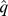) of LoFs as the number of LoF variants divided by the sample size. The histogram of the LoF frequencies of all genes is shown. Also shown are the estimated distributions of 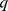 under the null (red, solid line) and the alternative (blue, dashed line) models, respectively.

Consider the *de novo* LoF mutations in 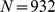 families [1]–[4]. These data reveal a total of 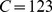 *de novo* LoF mutations across all genes, and 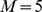 multiple-hit genes (at least 2 independent *de novo* LoF events per gene). Our goal is to find values of 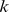 and 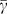 that best predict the observed counts 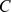 and 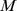 (Text S1). We assume that the relative risk of an ASD risk gene varies across 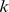 genes, with the average relative risk of the LoF mutations equal to 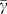. The mathematics of TADA reveal there is an inverse relationship between 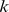 and 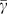 (Figure 3A, see Equation 27 in Text S1). For an alternative and more intuitive explanation of why these parameters have an inverse relationship, see the arguments in [2]. For any given value of 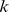, we can compute the expected number of multiple-hit genes; matching the expected with the observed value of 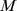, we estimate the the number of ASD risk genes is between 550 to 1000 (Figure 3B). In the next step, we use the HB model to estimate the most likely value of 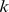 within this range, and the result is 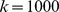 ASD risk genes, with the corresponding relative risk 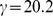 (see Text S1). These estimates are similar to published results using somewhat different methods [1], [2].

We examine evidence for the hypothesis that the population frequency of LoF mutations for ASD risk genes (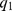) is lower than that for non-risk genes (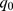) because mutations in ASD risk genes are under stronger negative selection than the average gene. These frequencies are of interest because they have a major influence on the power of association test [8]. We estimate 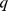 based on the number of LoF variants in the case-control data from the AASC [7] and the transmitted/nontransmitted data from 641 families (the transmission data are only available for a subset of the 932 families). To obtain the empirical distribution of 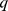 across all genes we first count the frequency of the LoF mutations in each gene (Figure 3C); we find a substantial number of genes with 0 LoFs. We next estimate the prior distributions of 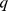 under the null and alternative models, respectively, using the HB model and find they provide a good fit to the observed data (Figure 3C, Figure S1). From these analyses the mean of 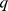 under 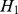, i.e. the average 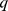 for ASD risk genes, is about 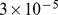, significantly smaller than that of non-risk genes, 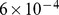 (see Text S1 for a description of how the HB model uses a mixture model to permit estimation of parameters specific to ASD risk genes without actually classifying genes as such.) Notably, while the empirical estimate of 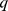 for most genes is 0 (thus not useful for inference), the value of 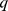 from the HB model is never equal to 0 due to smoothing.

Using the same procedures we also estimated these parameters for missense mutations that are probably damaging according to the PolyPhen prediction [9] (denoted as Mis3 mutations). Estimates reveal lower risk for these mutations, as expected, and lower 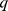 for ASD risk genes compared with non-ASD genes (Table S1).

### Power analysis by simulation

Equipped with estimates of the genetic parameters, we can simulate genetic data under the model and assess the performance of statistical methods. We compare performance of three tests: *De Novo*, as described in Section 2.1; TADA, described in Section 2.3; and a “Meta test”, which combines two tests, one based on *de novo* events and the other on inherited variants, via meta analysis. For the meta test we compute the *p*-value from data on inherited variants using a Fisher exact test, treating transmitted/untransmitted events as case-control data; and compute a *p*-value for *de novo* events using the *De Novo* test. Then these *p*-values are combined using Fisher's method. In all the simulations, different parameters are used to generate the data, yet TADA always uses the same set of parameters derived from the real data, as described previously. Thus these results establish the robustness of TADA under different parameter settings and thus, to some extent, how it should behave for real data.

Because TADA is a novel method, data were first simulated under the null hypothesis of no association to obtain the distribution of the TADA test statistic and its associated *p*-values. The results show that the test is well calibrated and type I error is properly controlled (Figure S2).

**Figure 4:**
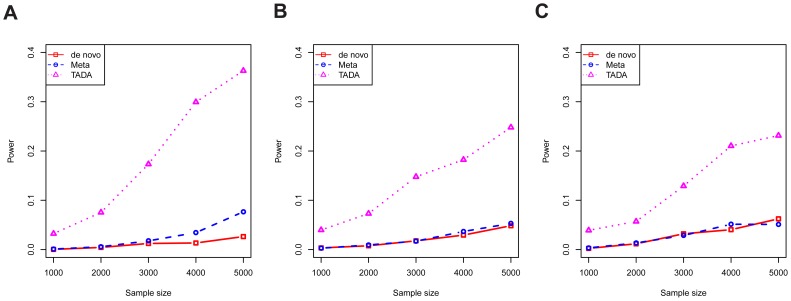
The power per gene of competing tests. The results of three tests are shown: novo (red), meta (blue), and TADA (purple). Results are shown for various values of 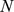, 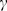 and 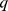 with type I error fixed at 0.001. Parameter values are chosen to cover plausible parameter values according to our model estimation: (A) 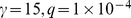; (B) 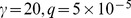; and (C) 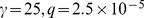.

Next, data were simulated under the alternative model, using different sample sizes and different combinations of the parameters 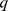 and 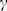, within the range of plausible values estimated in the previous section. This comprehensive simulation showed TADA has superior power relative to the other two tests (Figure S3). In Figure 4, we show a selected portion of the simulation results under the most likely scenarios, reflecting the trade-off between relative risks and allele frequencies, i.e. mutations with high risks are likely to exist in lower frequencies in the population. For a gene with typical parameter values (Figure 4B), the power of the TADA test, at 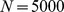, was about fivefold larger than that of the other two tests.

To assess the performance of the tests from a genome-wide analysis, we generated realistic simulated counts based on the estimated genetic parameters for ASD, namely average relative risk of 20 and 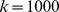 risk genes, among a total of 18,000 genes sequenced. We focus on false discovery rate (FDR), calibrating the empirical FDR to control at 10%, and estimated power as the number of true discoveries. Results confirmed the advantage of TADA (Figure S4A). For example, at 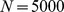, TADA identified more than 200 ASD risk genes at FDR below 10%, while the *De Novo* and Meta tests identify about 50 and 70 genes at this level of FDR, respectively (cf Figure 1). We performed additional simulations with somewhat different procedures to demonstrate the robustness of these findings. In one experiment, we simulated data under the average relative risk of 10, instead of 20, while TADA still uses the relative risk of 20. The power of all methods was significantly reduced, as expected, yet TADA still performed better than both *de novo* test and the simple meta-analysis (Figure S4B). In another experiment, the simulation procedure incorporated the possible dependency between the LoF frequency of a gene (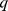) and its relative risk (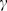), based on simple mutation-selection balance: the two were not sampled independently, but rather the frequency was inversely proportional to the risk (see Methods). Despite this change of simulation model, the results were virtually identical to those from earlier simulations (Figure S4C).

### Analysis of data to identify genes affecting the risk of ASD

**Figure 5:**
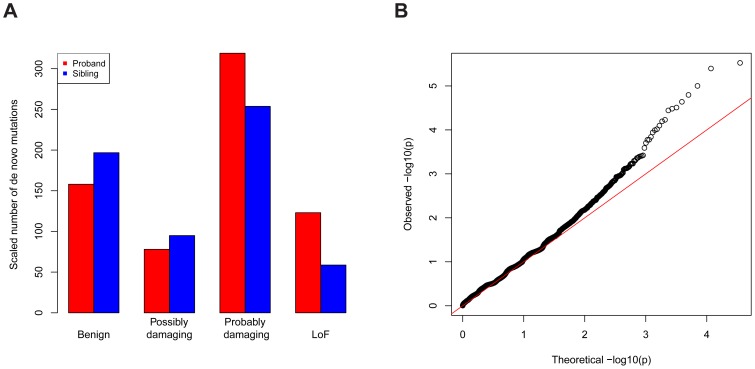
Application of TADA to the genetic data of ASD. (A) *De novo* LoF and “probably damaging” missense mutations are enriched in ASD probands (red) compared with unaffected siblings (blue), based on a comparison including all trio and quad families. The other types of missense mutations are not enriched. To make the numbers comparable, the number of mutations in siblings is scaled by a constant multiplier (214/124) so that the numbers of silent mutations is equal in probands and in siblings. The annotations of missense mutations are based on PolyPhen. (B) Q-Q plot (log. scale) of the 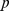 values for all genes in the ASD dataset based on a combined analysis of LoF and severe missense mutations.

The data we used were all reported *de novo* mutations from 932 ASD families [1]–[4]; transmitted mutations from 641 of these families; and case-control data from the AASC, consisting of 935 ASD subjects and 870 controls [7]. Each missense mutation was classified into a category of damage to the protein based on its predicted effect on the coding sequence using PolyPhen2 [9]: benign (Mis1); possibly damaging (Mis2); and probably damaging (Mis3). Note that *de novo* LoF mutations occurred at about two-fold enriched rate in the probands relative to the unaffected siblings (Figure 5A, Table S2). The rate for *de novo* Mis3 was also higher in probands than siblings, but the difference was not as striking. There is essentially no difference in probands and siblings for other types of mutations. We thus applied the TADA method to the LoF and Mis3 mutations.

The overall inflation of the results due to population stratification is negligible: a modified [7] genomic control factor [10] 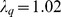 (see Text S1). There is significant enrichment of genes with low *p*-values compared with random expectation (Figure 5B): 244 genes have 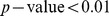, 64 more than expected under the null model. There is an intriguing coincidence in the excess of small *p*-values - namely that it is very similar to the excess number of genes with single-hit *de novo* LoF events in ASD subjects compared to their unaffected siblings [1]. Notably the large tail in the QQ plot is largely driven by the *de novo* LoF events, and appears to reflect true signal instead of inflation.

We control for the multiple hypothesis testing using the Benjamini-Hochberg procedure [11]. Fifteen genes meet the criteria of a False Discovery Rate less than 20% (Table 1, see Table S3 for the complete results). The list includes all five genes with two *de novo* LoF mutations, as well as several novel genes that are promising candidates for ASD based on existing evidence. For the novel predictions, the *p*-values from the *de novo* data alone are far from achieving genome-wide significance (the 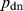 column in Table 1) and would be impossible to identify without combining the *de novo*, transmitted and case-control data.

**Table 1:** Top predicted ASD risk genes from the TADA analysis of combined ASD data (*de novo*, inherited and case-control).

	Loss of function (LoF)						
Gene	*De novo*	Transmitted	Nontransmitted	Case	Control	*p*_dn_	*p*_TADA(LoF)_
KATNAL2	2	1	0	4	0	3.1×10^−6^	2×10^−7^
CHD8	2	0	0	3	0	9.5×10^−5^	2.4×10^−6^
LMCD1	0	2	0	0	0	1	0.067
S100G	1	0	0	3	0	0.00042	1.6×10^−5^
DYRK1A	2	0	0	0	0	8.6×10^−6^	4.3×10^−6^
PPM1D	1	0	0	2	0	0.0032	0.00023
SCN2A	2	0	0	0	0	5.9×10^−5^	2.8×10^−5^
CUL3	1	0	0	3	0	0.004	0.00013
DEAF1	0	2	0	1	0	1	0.031
BANK1	0	1	0	4	0	1	0.0064
POGZ	2	0	0	0	0	3×10^−5^	1.4×10^−5^
WDR55	0	1	0	0	0	1	0.18
FAM91A1	1	0	0	0	0	0.0046	0.0019
COL25A1	1	0	0	5	0	0.0034	2.3×10^−5^

	Probably damaging (Mis3)					
Gene	*De novo*	Transmitted	Nontransmitted	Case	Control	*p*_TADA_
KATNAL2	0	2	3	4	5	1.5×10^−6^
CHD8	0	4	6	9	9	1.3×10^−5^
LMCD1	0	4	0	9	0	1.7×10^−5^
S100G	0	0	0	0	0	2.1×10^−5^
DYRK1A	0	0	4	4	1	5.6×10^−5^
PPM1D	0	0	0	2	0	7.9×10^−5^
SCN2A	1	8	7	5	5	8.4×10^−5^
CUL3	0	0	0	1	0	8.6×10^−5^
DEAF1	0	1	0	8	0	0.0001
BANK1	0	7	0	6	2	0.00011
POGZ	0	4	1	3	5	0.00012
WDR55	1	0	0	6	0	0.00012
FAM91A1	0	12	1	2	2	0.00016
COL25A1	0	5	3	4	4	0.00016

The 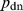 column shows the *p*-values using the *De Novo Test* from the *de novo* LoF mutations alone. The 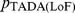 column shows the *p*-values from the TADA test using all LoF data. The 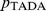 column shows the *p*-values from the TADA test using both LoF and Mis3 data. The star symbols mark the double-hit genes that were reported in earlier publications. C1orf95 also has *q*-value<.2, however this signal is based entirely on 11 identical Mis3 variants in cases and 0 in controls. This allele is common in African populations [40]. While the AASC sample is of European ancestry, a portion of it, largely from Portugal, carries some sub-Saharan alleles [7]. Thus, this signal is likely due to population substructure. Similarly, the 3 LoF variants seen in S100G are copies of a splice variant that is common in African populations, so this result should be viewed with caution.

The results of TADA generally depend on the estimates of the mutation rates of the genes, as well as the Bayesian prior parameters of the model. We perform additional analyses to study how sensitive the results are to these parameters. Based on our findings, we choose several genes from Table 1 for this investigation. Although the error of mutation rate estimation is likely small [1], we vary the mutation rate of each gene: from 1/2 of the estimated rate to twice the rate. As expected, the *p*-value increases as the mutation rate increases, although overall the impact is modest (Figure S5A). Next we vary the Bayesian prior parameter, 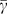, which represents the average relative risk over all risk genes, from 10 to 20. The *p*-values from TADA are even less sensitive to this parameter (Figure S5B).

## Discussion

For disorders like ASD, recent results show that detection of *de novo* LoF events can be a powerful means of discovering novel risk genes [1]–[4]. Yet *de novo* events are relatively rare, roughly one per exome, and *de novo* LoF events even more so, and thus many families must be assessed to identify multiple *de novo* LoF events in the same gene. To make the most of this experimental design, we develop a new statistical approach, TADA, that utilizes both transmitted and *de novo* variants from nuclear families and case-control data to determine genetic association. TADA builds on the simple multiplicity test, which relies on recurrent *de novo* events, but it creates a full analytical framework to incorporate all of the information on the distribution of rare variation. The result is a test with greater power. Our test achieves its good performance properties by providing an analytic framework that links the observed pattern of *de novo* mutations with the underlying genetic parameters, such as the relative risk conveyed by such mutations. In addition to analyzing data for novel gene discovery, this framework can be used to analyze the power of a test and predict the required sample size to attain sufficient power for future investigations. Moreover, by using empirical Bayes methods, TADA refines estimates of allele frequencies of the damaging mutations by using the full genome to estimate these quantities. This approach increases the information in the transmitted variants in each gene considerably and yet maintains good control of false discoveries.

Association studies evaluating cases and controls have been a common design for identifying variation affecting risk for complex diseases. It has proven successful for identifying common variation affecting risk, after sufficient samples had been amassed to ensure variation having modest impact on risk could be detected [12]. Common variants surely play a role in ASD [13], [14], but the effect sizes are small [15] and it will be challenging to detect individually-significant SNPs. Indeed virtually every discovery for ASD risk genes traces to rare and *de novo* variants [1]–[4], [16]–[20].

As the cost of sequencing drops, genetic research increasingly focused on the role of rare variants in complex diseases such as ASD, but the sample size has been limited and so has the yield of such studies. For a sample of nearly 1000 ASD case and well matched controls the ARRA ASD sequencing consortium (AASC) found no significant associations [7], except for variation acting recessively [6]. These results comport with studies of other disorders and suggest that large sample sizes will be required to achieve good power in rare variant association studies [21]. Arguably a fundamental difficulty is that most of the mutations with large effects tend to be under strong negative selection, existing at very low frequencies in the population [22]. Variants that occur with greater frequency often have smaller effect on the phenotype, reducing the power of gene-based test statistics.

Our analysis provides insight into some advantages of *de novo* over case-control studies, especially for LoF events. The *de novo* test gains power because the mutation rate for genes can be estimated accurately from supplementary sources, and need not be estimated as part of the statistical procedure. Because of the low mutation rate, the number of *de novo* LoF events expected by chance is very small, and thus we could attach high statistical significance to any gene with more than one independent LoF mutation. While a single *de novo* LoF event is certainly not definitive evidence, it can put a gene on the short list as a risk gene – for ASD, it is more likely than not an ASD risk gene. In contrast, for case-control data, we require an estimate of the allele frequency 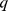 under the null hypothesis. When the mutant allele is very rare (as for ASD risk genes), a very large sample is required to ensure that this frequency is indeed small.

Another feature of observed *de novo* mutations is that they have not been subject to the force of purifying selection, which plays a key role in shaping the pattern of standing variation. Therefore it is likely that *de novo* mutations, especially LoF mutations, have stochastically larger effect sizes than rare variation transmitted for generations, because selection tends to drive down allele frequencies of variants having large effects on reproductive success. Moreover, allele frequency is inversely tied to power, critical for any experimental design. Therefore studies utilizing *de novo* variation can have distinct advantages, in terms of power, relative to those that do not.

By simulations we demonstrate that the power of TADA is higher than tests based solely on *de novo* events or standard meta-analysis that combines *p*-values from *de novo* and inherited data (transmission or case/control). There are two explanations for this gain of power. First, TADA's hierarchical model uses the information in the case-control (or transmission) data more efficiently than the standard hypergeometric or trend test. One important property of LoF mutations, compared to less severe functional variants, is their rarity in the population (Figure 3C). TADA, which is similar in spirit to a Poisson test of rare events, is able to exploit the rarity of these damaging events by estimating the distribution of LoF alleles across the exome (see Figure S1B), whereas the other methods cannot. Second, because damaging *de novo* mutations are rare, most genes will not harbor them even when thousands of cases have been sequenced. For such genes, using Fisher's method to combine the *de novo p*-value, which will be close to 1, with the *p*-value from the case-control data penalizes the overall test statistic. In contrast, the Bayesian approach uses *de novo* events when they are informative and disregards the *de novo* data when they are uninformative; the Bayes factor from *de novo* in such cases would be close to 1, making little contribution to the gene's total Bayes factor.

We estimate that there are about 1,000 ASD risk genes with average relative risk about 20. In a recent paper using the same *de novo* data, the number of ASD risk genes (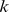) was estimated at 370 [4]. In that paper, the expected number of genes with recurrent LoF events was derived as a function of 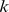, and equating it to 5 (the observed number), produced the solution that 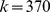. The analysis made the implicit assumption that all ASD risk genes are equally likely to sustain multiple *de novo* LoF events. In Text S1 we show, using Jensen's Inequality, that the non-uniform distribution of the mutation rates and the relative risks among the ASD risk genes leads to a significant under-estimation of 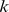, explaining the discrepancy between our results and those of Iossifov et al. [4].

When applied to ASD data, TADA predicts a number of novel ASD risk genes (Table 1), as well as supporting results for known ASD risk genes. For some of the newly implicated genes it is straightforward to garner other supporting evidence for their role in ASD. *S100G* is a downstream target of *CHD8*, a key transcriptional regulator often disrupted in ASD subjects [23]. *CUL3* plays a critical role in neurodevelopment [24], [25] and in particular regulates synaptic functions [26]. A recent study identified an additional *de novo* protein-changing mutation in *CUL3* in ASD probands [27], replicating our finding here. *COL25A1*, a brain-specific collagen, was implicated in risks for Alzheimer's disease [28] and antisocial personality disorder [29].

Inspection of other genes slightly below our chosen FDR threshold reveals several more interesting genes that likely play some role in ASD (all ranked among the top 25, see Table S3). *TBR1*, a transcription factor critical in brain development, regulates several known ASD risk genes [30]. A recent study has identified recurrent *de novo* disruptive mutations in *TBR1* in ASD subjects [23]. *MED13L*, a component of the Mediator Complex, is intriguing because of its role in Rb/E2F control of cell growth [31] and the fact that RB/E2F plays a key role in neurogenesis [32] and neuronal migration [33]. Recently *MED13L* has been associated with risk for schizophrenia [34]. *NFIA* is a member of the NFI transcription factor family, thought to have a neuroprotective role [35], and *NFIA*-knockout mice display profound defects in brain development [36].

Genotyping/sequencing errors can introduce biases in data analyses, especially those for family data [37], [38] and for combining data across multiple heterogeneous studies [39]. Our analyses are likely robust to these possible biases because the variant calls were all carefully evaluated: (i) all *de novo* mutations described previously [1]–[4] and analyzed here, a total of 122 LoF and 314 damaging missense mutations, have been validated by previous studies; (ii) the case-control data have been carefully harmonized to minimize batch effects by using stringent quality control filters [7]; and (iii) for the case-control data, all variant calls in two genes (CHD8 and SCN2A) have been evaluated by Sanger sequencing and 20 out of 20 validate, further supporting the quality of the variant calls in the case-control data. When the sensitivity of calling minor variants is low (under-calling), this may create an under-transmission bias in family-based test statistics; however, TADA is effectively a one-sided test of the adverse effect of the minor allele. As such, TADA is only powered to detect risk variants that are over-transmitted and thus bias due to under-transmission is not a significant concern. Nonetheless, data quality is always an important concern, and can change over time in subtle ways [37], [38], making high-quality filters and validation of *de novo* events critical for good data analyses. It is possible that TADA would benefit by modeling measurement errors and this will be a topic for future research, when the error structure in the data is better understood.

While much of our focus has been on ASD data and the genetic architecture of ASD, TADA has utility beyond the genetics of ASD. For example, we would expect TADA to be useful for gene discovery by the analysis of data from any genetic disorder or disease for which *de novo* mutations play a substantive role in risk. Early onset diseases and disorders are obvious candidates for the use of TADA, as are disorders such as schizophrenia and congenital heart disease. Indeed there are a plethora of human diseases for which *de novo* mutations account for at least a small fraction of risk, even diseases that onset in mid-life such as cardiovascular disease. Because TADA is based on a general theoretical framework for combining rare variation found in exons of genes, we predict that its logic can have even broader applications than simply the analysis of single genes for their association with disease.

## Methods

### Sequence data

We combined exome sequence data from four recent studies of ASD, covering 932 families [1]–[4]. Detailed information about study design, including family structure (simplex versus multiplex), ascertainment, and DNA source (blood versus cell line), can be found in the Supplements of these papers. The *de novo* mutations, including both single nucleotide variants (SNVs) and indels, were identified as described in the original papers. The transmitted and non-transmitted variants were extracted from 641 of these families (see Text S1 for details on data processing). We excluded all common variants from the analysis, defined as those present at 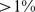 population frequency in the Exome Sequencing Project (ESP) controls and/or the 1,282 parents [40]. Only SNVs were called for the transmission data, indels were not identified. We also included case/control data from the ARRA ASD Sequencing Consortium, consisting of 935 ASD subjects of European ancestry and 870 controls of ancestry similar to cases, selected from the NIMH repository (see complete information on study design in the supplement of Liu et al [7]). The SNVs and indels in the case/control were called as described in [7]. Each mutation/variant in the combined data was classified into different categories, based on its predicted effect on the protein function, according to the program PolyPhen2 [9]. In this study we focused on (1) LoF mutations, defined as nonsense mutations, mutations in splice sites or frameshift indels; and (2) mutations classified as “probably damaging” to protein function by PolyPhen2 (Mis3). We also removed all genes with more than 10 LoF events in the control samples (166 genes in total) from the analysis, as these genes are unlikely to be related to ASD.

### Mutation rate estimation

For each gene, the total rate of base pair substitutions was estimated using a probability model taking the gene length and base content into account [1]. To estimate the rate of a specific type of mutation (LoF or Mis3) of a gene, we multiplied the gene-level mutation rate and the proportion of that type of mutation. The proportion of LoF or Mis3 mutations was estimated from the data of unaffected siblings in the ASD families (Table S2). In these siblings, there were 461 single-nucleotide variants (SNVs) and 34 LoF variants, thus the LoF fraction was 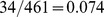. Similarly the Mis3 fraction was calculated as 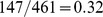.

### TADA model and the statistical test

**Figure 6:**
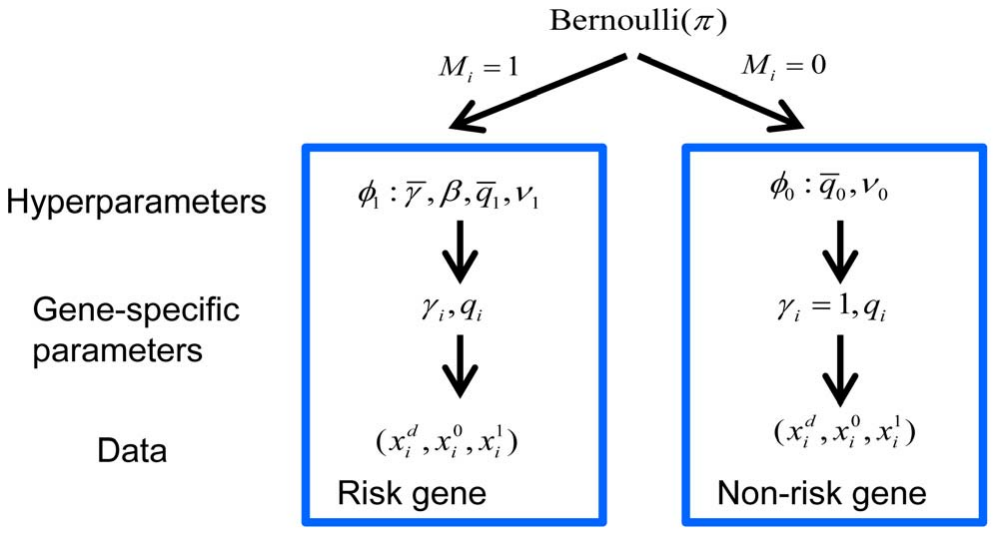
Bayesian hierarchical model of TADA. A fraction 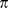 of the genes are associated with the phenotype under investigation and follow model 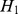, and the remainder follow model 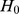. The prior distribution of gene-specific parameters, relative risk (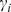) and allele frequency (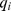), can vary under the competing models, 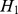 or 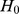. Priors are specified by the hyperparameters, 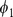 and 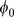, respectively, which are estimated from the data. Counts of events for the *i*-th gene follow a Poisson distribution, parameterized by 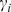 and 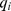 under 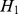, and 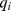 under 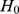.

Two hypotheses were compared, 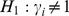 versus 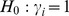, for each gene. For most genes, the number of LoF mutations either transmitted or not (or in cases and controls) was generally very small and often 0, leading to a naive estimate of 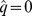 and creating a challenge for a likelihood-based test. To refine inference we took an Empirical Bayes approach and developed a hierarchical Bayes model for the data (Figure 6). We estimated the prior parameters in the model by maximizing the marginal likelihood. The hierarchical model assumed a fraction 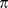 of the genes was associated with the disorder (model 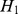) and the remaining fraction followed the null model (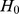). Under 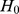, we assumed 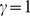 for all genes and 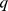 followed a Gamma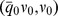 distribution (we parameterized the distribution so that its mean was 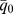). The scaling parameter of the Gamma distribution (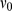) played the role of a precision parameter or pseudo count; the bigger 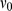 the more similar 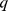 was estimated to be across genes. Under 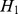, we assumed 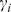 of the *i*-th risk gene follows a 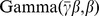 distribution and 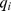 follows a 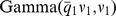 distribution.

Let 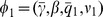 be the prior parameters of 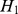, and 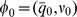 be those of 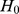 (they are also called hyperparameters). The counts for the *i*-th gene, 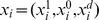, follow Poisson distributions parameterized by 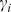 (1 for non-risk genes) and 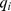, as defined in Equation 1.

The marginal likelihood of the *i*-th gene under either model, 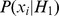 and 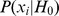, is given by:
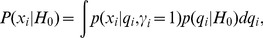
(4)

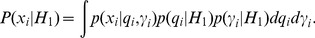
(5)The marginal likelihood of all the data, as a function of the hyperparameters 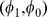, is
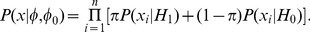
(6)We assume the proportion of risk genes, 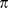, is known (in our analysis of ASD data, this is obtained by the estimated value of 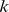, the number of ASD risk genes, see Section 2.4). The hyperparameters can then be found by maximizing this marginal likelihood function. Once we have the estimated values of 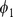 and 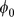, we compute the Bayes factor of any gene:
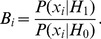
(7)The *p*-values of the observed Bayes factors are calculated by sampling the null distribution according to Equation 1 (see Text S1).

### TADA for multiple types of mutations

When analyzing multiple types of mutations (LoF and Mis3 in our analysis of ASD data), we assumed the data for each type of mutation were independent of each other, and hence we estimated the prior parameters for each type of mutation separately. The Bayes factor of a gene is defined as the product of the Bayes factor for each type of mutation. For these ASD data, the Mis3 mutations are likely to be a mixture of those causing protein-damaging changes and those having no real effects on the protein function. We thus computed the joint Bayes factor of the gene using this equation:
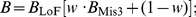
(8)we used 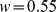 in our ASD analysis (see Text S1).

### Simulation procedure

Our simulation procedure generated data using the estimated genetic parameters of the LoF mutations of the ASD risk genes (Text S1). For our initial simulations, we compared the power of several statistical tests, at the single gene level, under various combinations of parameter values. We set the mutation rate as the mean mutation rate of the LoF mutations of all human genes, 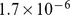. The parameters 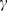 and 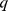 were chosen according to their estimated mean values: 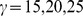, and 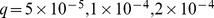. We compared the power of the three tests under type I error 0.001.

For the second set of simulations, we assessed the performance of the three tests in the genomewide setting. Specifically, from among 18,000 genes in the human genome, we first randomly sampled 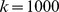 risk genes and the rest were assumed to be unrelated to disease (we used the estimated mutation rates of all genes to make this simulation realistic). For a risk gene and a LoF mutation, the effect size parameter 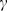 was sampled from the distribution 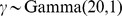. Its population frequency parameter 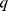 was sampled from the distribution, 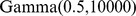. For a non-causal gene, its relative risk 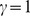, and the frequency parameter 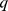 was sampled from the distribution 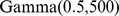. The simulation procedure then generated, for each gene, the number of *de novo* mutations (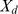), the number of transmitted variants (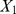) and the number of nontransmitted variants (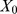), according to Equation 1.

We ran the three statistical tests, as described in the text, on the simulated data from all genes. At various significance levels, we calculated the number of true discoveries (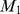), i.e. the number of diseases genes whose test statistic reached significance level . We chose the value of  so that FDR is less than 0.1, and reported 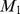 at this value of  (see Text S1 for our procedure for controlling FDR in the simulations.)

In additional simulations, we varied the basic procedure just described. In one setting, the average relative risk was set to 10 instead of 20, i.e., 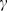 of a risk gene was sampled from the distribution 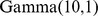. In another setting, instead of sampling 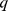 and 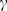 of each risk gene independently, we modeled the two as dependent. Specifically, for the *i*-th risk gene, let 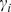 and 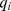 be the relative risk and the LoF frequency, respectively. First sample 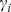 from 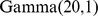, then determine 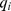 according to a simple mutation-selection balance: 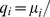, in which 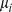 is the mutation rate and  is a constant. To determine the value of , we plugged in the mean values of 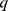, 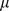 and 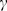 in the above equation and solve 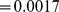.

### Software

TADA software is available as an R package at http://wpicr.wpic.pitt.edu/WPICCompGen/. The package also includes TADA-Denovo, the simplified version of TADA, that analyzes only *de novo* data.

## Supporting Information

Figure S1Bayesian estimation of the frequency parameter 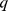. (A) The observed LoF counts (red) of all genes, vs. the simulated counts (blue). For simulation of one gene, we first sample 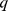 from the estimated prior distribution of 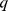 under 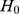, and then generate the count data under this 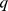 according to the Poisson model (Equation 1 of the text). The procedure is repeated for all genes, and the resulting barplot is provided along with the distribution of the observed data. Note that we did not use the distribution 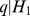, as most of the genes are not disease-related. (B) The Bayesian hierarchical model estimation of the allele frequency 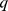 of LoF variants. The blue circles show the observed frequencies of 10 different genes, which are also maximum likelihood estimates (MLE). The red circle shows the average 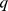 over all genes (prior mean). The Bayesian posterior mean estimates are the weighted average of the MLE and the prior mean (the intersection of the dashed line and solid lines), with weight 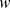 (0.20 in this example).(TIF)

Figure S2Typical Q-Q plots under the null distribution of the TADA test statistic. We simulate 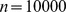 genes under the null model, with mutation parameter 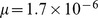 (the mean LoF mutation rate of all human genes), and 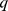 varying from from 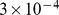 to 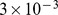 (the average 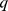 of non-autism genes is about 0.001) and number of family trios (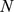) varying from 1000 to 3000. The TADA model is applied to each of 9 simulated datasets to obtain the *p*-values and resulting Q-Q plots. Although there is normal variation in these samples, most follow the expected null distribution fairly closely.(TIF)

Figure S3The power of the *de novo* test (red), the meta test (blue) and TADA (purple) at type I error 0.001, under various values of 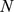, 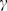 and 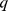.(TIF)

Figure S4The number of discovered disease genes as a function of sample size at FDR equal to 10%. We compare power for a test relying on only *de novo* events (*De novo Test*, red), a test combining *p*-values from *de novo* and transmitted data by Fisher's method (Meta test, blue), and the joint likelihood-based analysis (TADA test, purple). Results from three different simulations are shown. (A) Simulation using the estimated ASD parameters (the average relative risk 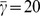). (B) Simulation assuming 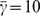. (C) Simulation under the inverse-relationship between the LoF frequency (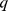) and the relative risk (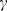) for each risk gene.(TIF)

Figure S5Sensitivity analysis of TADA for four selected genes. (A) For each gene, suppose 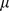 is its (estimated) mutation rate, we let TADA use a different rate, ranging from 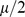 to 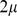, and the resulting *p*-values are shown. (B) We vary the prior parameter 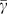 (the average relative risk of all risk genes) of TADA from 10 to 20, and compute the TADA *p*-values.(TIF)

Table S1 Parameters from Hierarchical Bayes estimation. The LoF and damaging missense (Mis3) mutations of ASD genes have high relative risks, and appear to be under stronger purifying selection than non-ASD genes.
(PDF)

Table S2 The statistics of the *de novo* mutations in autism probands and unaffected siblings. The missense labels are based on predictions from PolyPhen2. Missense1–3 correspond to “benign”, “possibly damaging” and “probably damaging” mutations, respectively. The last row is the counts of frameshift indels.
(PDF)

Table S3The complete prediction results of TADA. The “mut.rate” column shows the estimated mutation rate of the genes. For each of the two types of mutations, LoF and mis3 (severely damaging), five counts are shown, including the number of *de novo* mutations, the numbers of transmitted and non-transmitted variants, and the number of variants in cases and controls. The 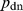 column shows the *p*-values using the *De Novo Test* from the *de novo* LoF mutations alone. The 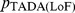 column shows the *p*-values from the TADA test using all LoF data. The 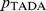 column shows the *p*-values from the TADA test using both LoF and Mis3 data. The last column shows the *q*-value of 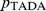 after Benjamini-Hochberg correction of multiple testing.(XLSX)

Text S1 Supplementary methods explaining the details of TADA, and our analysis of ASD data.
(PDF)
