# Supplementary figures and images for: Integrated Model of De Novo and Inherited Genetic Variants Yields Greater Power to Identify Risk Genes
Source: PLoS Genet. 2013 Aug 15;9(8):e1003671. doi: 10.1371/journal.pgen.1003671 (PMC3744441; doi:10.1371/journal.pgen.1003671)

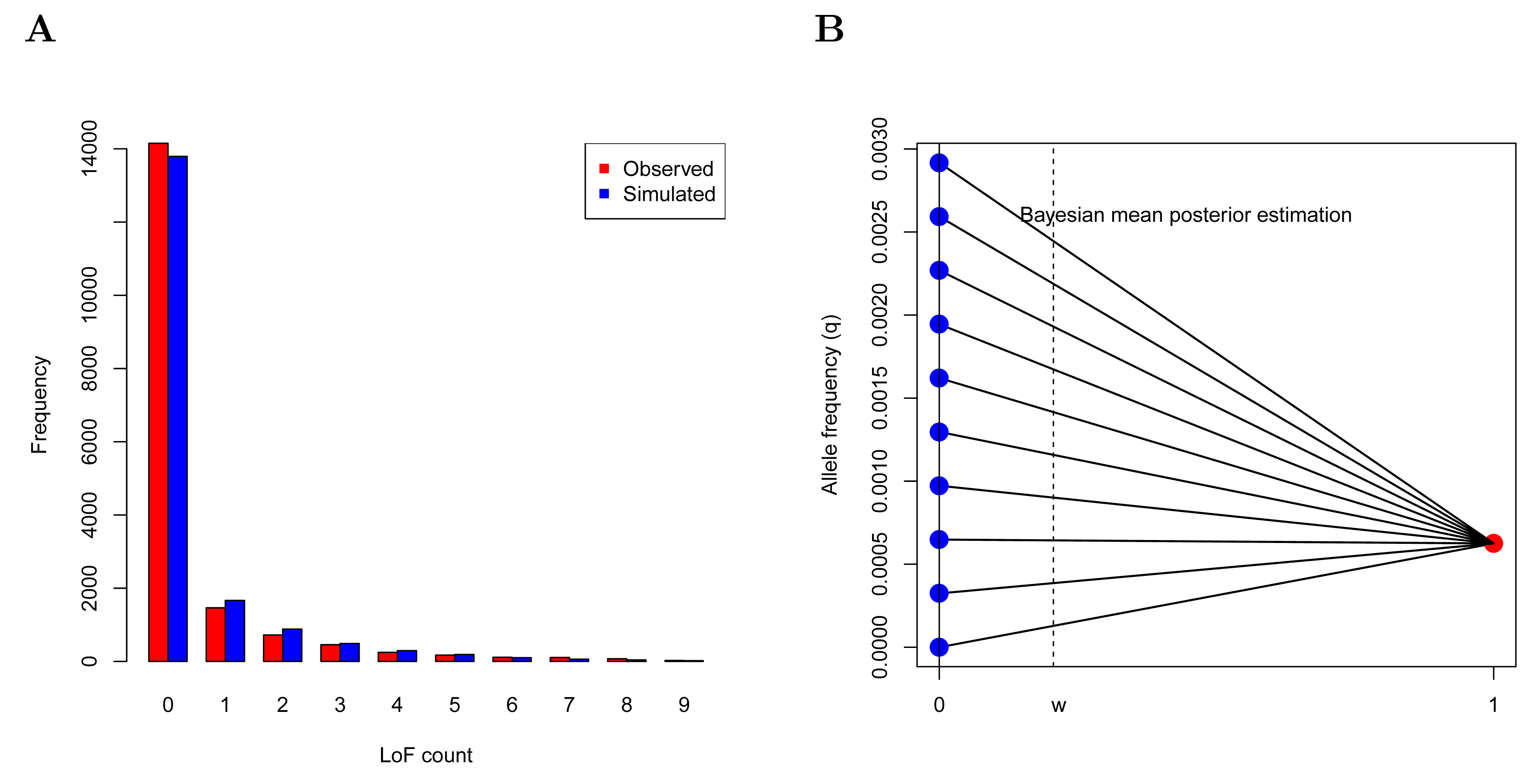

Supplement: Figure S1 — Bayesian estimation of the frequency parameter . (A) The observed LoF counts (red) of all genes, vs. the simulated counts (blue). For simulation of one gene, we first sample from the estimated prior distribution of under , and then generate the count data under this according to the Poisson model (Equation 1 of the text). The procedure is repeated for all genes, and the resulting barplot is provided along with the distribution of the observed data. Note that we did not use the distribution , as most of the genes are not disease-related. (B) The Bayesian hierarchical model estimation of the allele frequency of LoF variants. The blue circles show the observed frequencies of 10 different genes, which are also maximum likelihood estimates (MLE). The red circle shows the average over all genes (prior mean). The Bayesian posterior mean estimates are the weighted average of the MLE and the prior mean (the intersection of the dashed line and solid lines), with weight (0.20 in this example). (TIF) [file pgen.1003671.s001.tif]

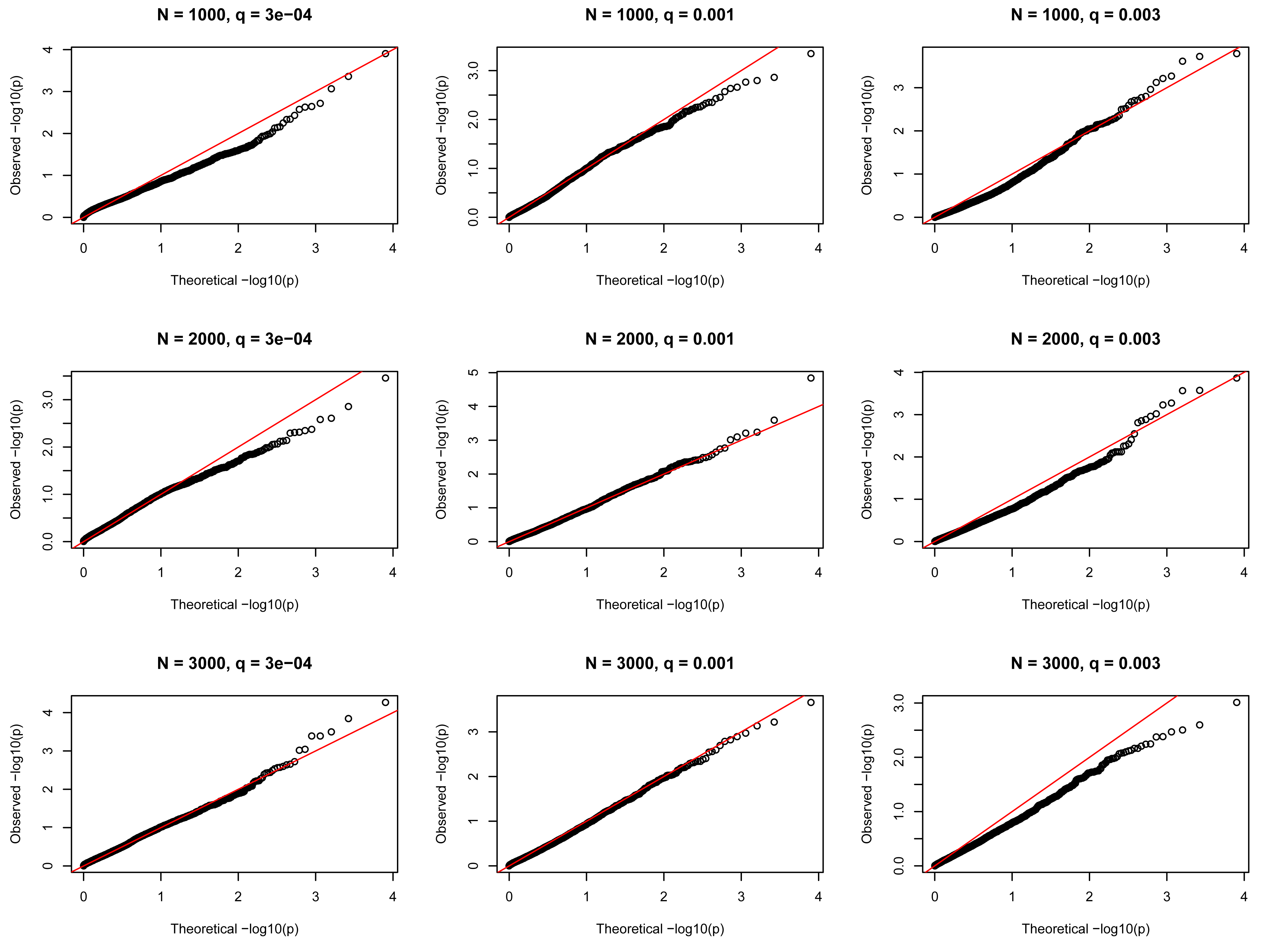

Supplement: Figure S2 — Typical Q-Q plots under the null distribution of the TADA test statistic. We simulate genes under the null model, with mutation parameter (the mean LoF mutation rate of all human genes), and varying from from to (the average of non-autism genes is about 0.001) and number of family trios () varying from 1000 to 3000. The TADA model is applied to each of 9 simulated datasets to obtain the p-values and resulting Q-Q plots. Although there is normal variation in these samples, most follow the expected null distribution fairly closely. (TIF) [file pgen.1003671.s002.tif]

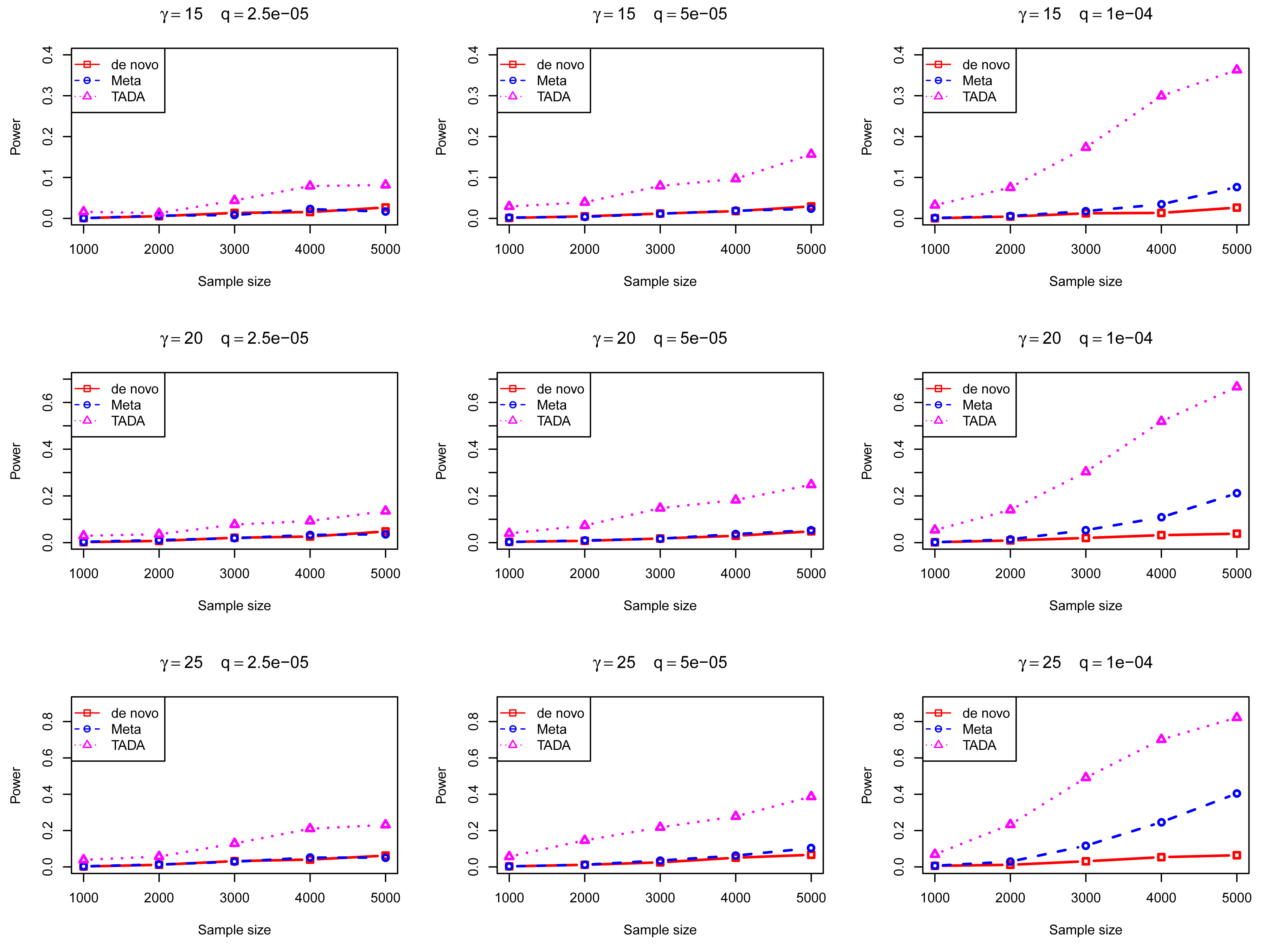

Supplement: Figure S3 — The power of the de novo test (red), the meta test (blue) and TADA (purple) at type I error 0.001, under various values of , and . (TIF) [file pgen.1003671.s003.tif]

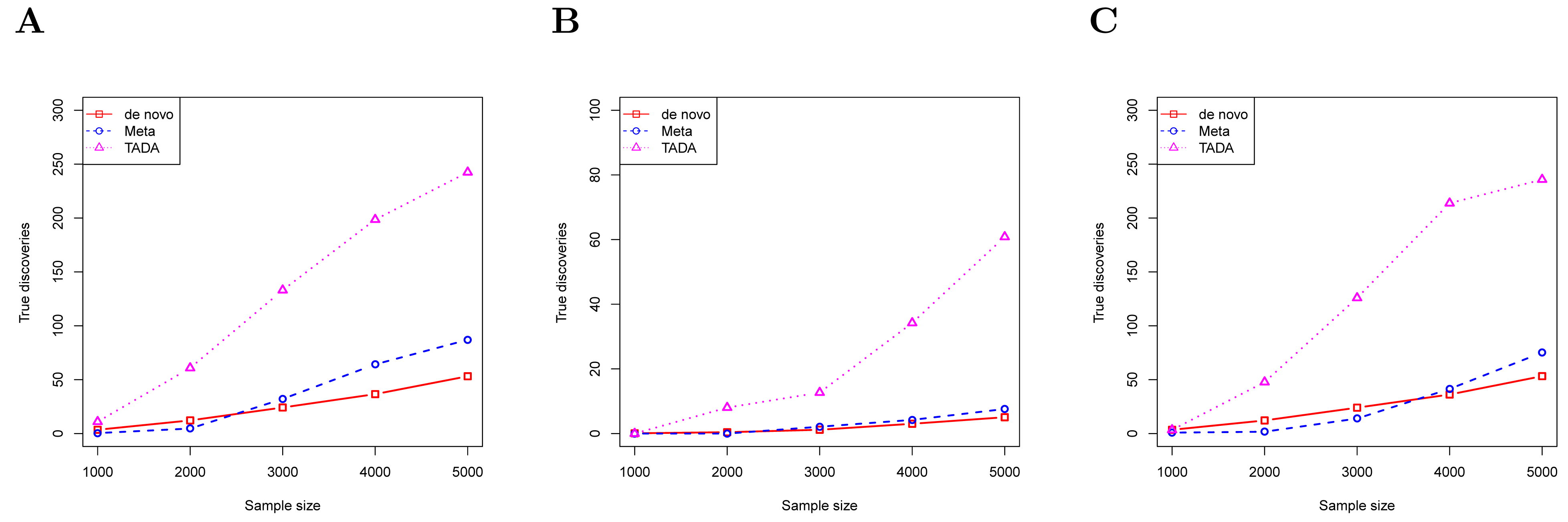

Supplement: Figure S4 — The number of discovered disease genes as a function of sample size at FDR equal to 10%. We compare power for a test relying on only de novo events (De novo Test, red), a test combining p-values from de novo and transmitted data by Fisher's method (Meta test, blue), and the joint likelihood-based analysis (TADA test, purple). Results from three different simulations are shown. (A) Simulation using the estimated ASD parameters (the average relative risk ). (B) Simulation assuming . (C) Simulation under the inverse-relationship between the LoF frequency () and the relative risk () for each risk gene. (TIF) [file pgen.1003671.s004.tif]

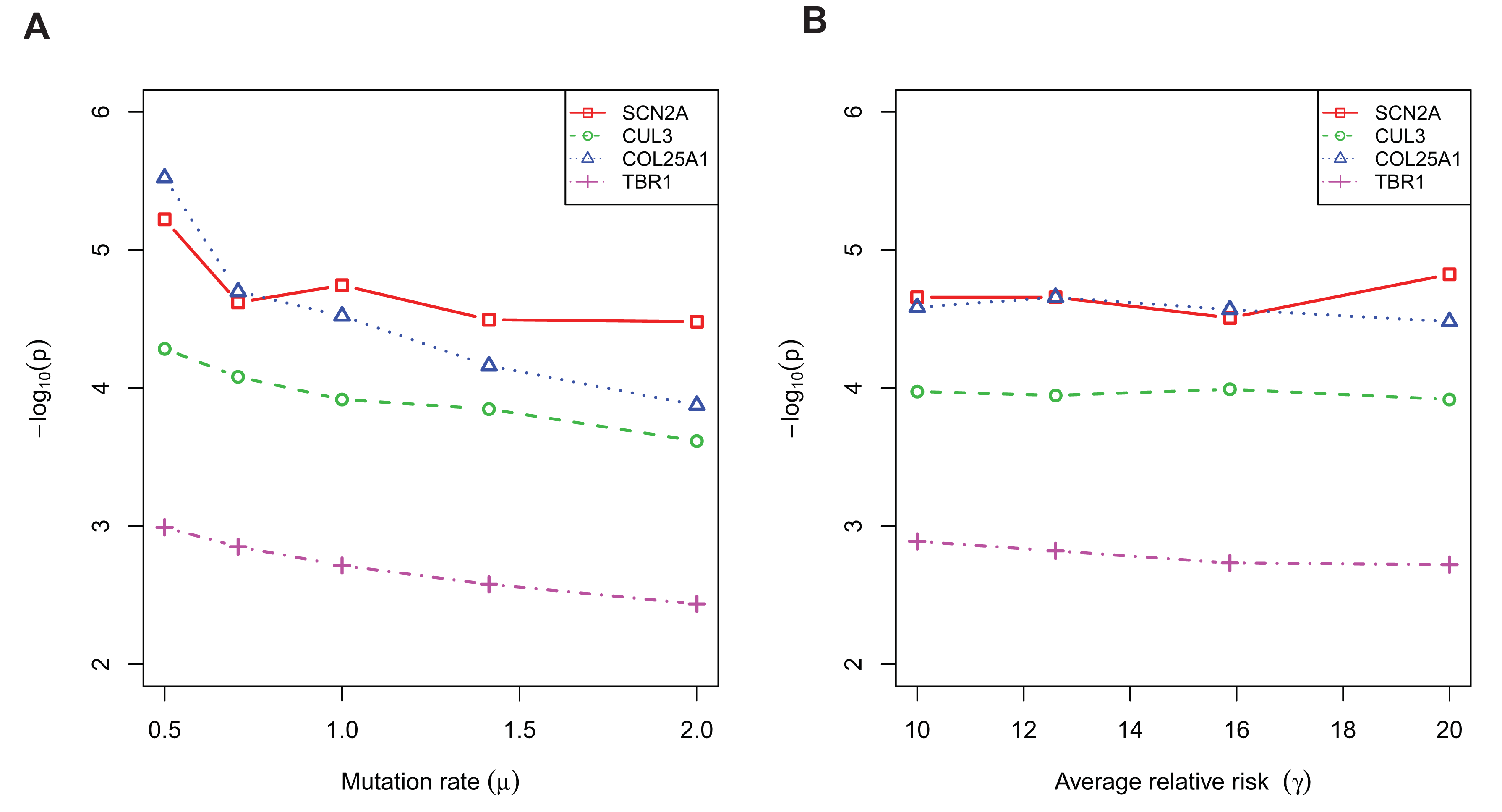

Supplement: Figure S5 — Sensitivity analysis of TADA for four selected genes. (A) For each gene, suppose is its (estimated) mutation rate, we let TADA use a different rate, ranging from to , and the resulting p-values are shown. (B) We vary the prior parameter (the average relative risk of all risk genes) of TADA from 10 to 20, and compute the TADA p-values. (TIF) [file pgen.1003671.s005.tif]
