# Supplementary material for: Integrated Model of De Novo and Inherited Genetic Variants Yields Greater Power to Identify Risk Genes
Source: PLoS Genet. 2013 Aug 15;9(8):e1003671. doi: 10.1371/journal.pgen.1003671 (PMC3744441; doi:10.1371/journal.pgen.1003671)
Supplement: Table S1 — Parameters from Hierarchical Bayes estimation. The LoF and damaging missense (Mis3) mutations of ASD genes have high relative risks, and appear to be under stronger purifying selection than non-ASD genes. (PDF) [file pgen.1003671.s006.pdf]

Parameter	LoF	Mis3
$\bar{\gamma}$	20.2	4.7
β	1	1
$\bar{q}_1$	3.0×10^{-5}	6.0×10^{-4}
ν_1	10000	2000
$\bar{q}_0$	6.2×10^{-4}	3.7×10^{-3}
ν_0	800	206

Table 1: Parameters from Hierarchical Bayes estimation. The LoF and damaging missense (Mis3) mutations of ASD genes have high relative risks, and appear to be under stronger purifying selection than non-ASD genes.
