# Supplementary material for: Integrated Model of De Novo and Inherited Genetic Variants Yields Greater Power to Identify Risk Genes
Source: PLoS Genet. 2013 Aug 15;9(8):e1003671. doi: 10.1371/journal.pgen.1003671 (PMC3744441; doi:10.1371/journal.pgen.1003671)
Supplement: Table S2 — The statistics of the de novo mutations in autism probands and unaffected siblings. The missense labels are based on predictions from PolyPhen2. Missense1–3 correspond to “benign”, “possibly damaging” and “probably damaging” mutations, respectively. The last row is the counts of frameshift indels. (PDF) [file pgen.1003671.s007.pdf]

Type	Proband	Sibling
Silent	214	124
Missense1	158	114
Missense2	78	55
Missense3	319	147
Nonsense	51	16
Splice	18	3
Total SNV	842	461
Frameshift	54	15

Table 2: The statistics of the *de novo* mutations in autism probands and unaffected siblings. The missense labels are based on predictions from PolyPhen2. Missense1-3 correspond to “benign”, “possibly damaging” and “probably damaging” mutations, respectively. The last row is the counts of frameshift indels.
