# Supplementary material for: Integrated Model of De Novo and Inherited Genetic Variants Yields Greater Power to Identify Risk Genes
Source: PLoS Genet. 2013 Aug 15;9(8):e1003671. doi: 10.1371/journal.pgen.1003671 (PMC3744441; doi:10.1371/journal.pgen.1003671)
Supplement: Text S1 — Supplementary methods explaining the details of TADA, and our analysis of ASD data. (PDF) [file pgen.1003671.s009.pdf]

Supplementary Methods: Integrated model of *de novo* and inherited genetic variants yields greater power to identify risk genes

Contents

1 Probabilistic model of *de novo* mutations and inherited variations in family data 2

2 The impact of mutation rates on the Multiplicity Test 3

3 Bayesian inference of risk genes 3

4 Estimation of the prior parameters by the Hierarchical Bayes method 5

5 TADA for multiple types of mutations 5

6 Method of moment estimation of the number of ASD genes and their relative risks 6

7 Comparison with an earlier estimate of the number of ASD genes 7

8 Parameterization in the analysis of ASD data 8

9 Controlling FDR in simulations 9

10 Genomic control for TADA 10

11 Variant Calling 10

1 Probabilistic model of *de novo* mutations and inherited variations in family data

Given a trio of normal parents and an affected offspring, there are four primary genotype combinations (Figure 2 in the main text). The informative cases are the *de novo* mutations, nontransmitted and transmitted variants (the second, third and fourth cases of Figure 2, respectively). Risk genes are more likely to carry *de novo* mutations than non-risk genes, and also are more likely to display an imbalance of transmitted vs. nontransmitted variants (the basis of transmission disequilibrium test, or TDT). We use p_d, p_{nt} and p_t, to denote the probability and X_d, X_{nt} and X_t the count of events of *de novo*, not-transmitted and transmitted events, respectively, out of N_d families. For mutations with strong deleterious effects, all three probabilities are small. Hence we model the counts using Poisson distributions: $X_d \sim \text{Pois}(N_d p_d)$, and so forth for X_{nt} and X_t. Let x be the genotype (AA or Aa) and y be the phenotype of an individual (Disease or Unaffected). The probability of *de novo* mutations given the trio is:

$$p_d = P(x_p = AA/AA, x_c = Aa | y_p = UU, y_c = D) = \frac{P(x_p = AA/AA, x_c = Aa, y_c = D | y_p = UU)}{P(y_c = D | y_p = UU)}, \quad (1)$$

where the subscripts p and c represent the parents and the child respectively. To compute the denominator, we sum over all the possible genotypes of the parent and child:

$$P(y_c = D | y_p = UU) = \sum_{x_p, x_c} P(x_p | y_p = UU) P(x_c | x_p) P(y_c = D | x_c). \quad (2)$$

The genotype probabilities (the first two terms) and the phenotype probabilities (the last term) of the four cases are shown in Figure 2. Plugging in the relevant terms into Equation 1, we have:

$$p_d = \frac{2(1-q)\mu\gamma}{(1-q)(1-2\mu) + 2(1-q)\mu\gamma + q(1-\mu) + q(1+\mu)\gamma}. \quad (3)$$

We focus on rare variants, hence q is typically less than 1%. The *de novo* mutation rate is generally very low, on the order of 1×10^{-5} to 1×10^{-6} for LoF or damaging missense mutations. We thus have the approximation: $p_d \approx 2\mu\gamma$. Following the same logic, it is easy to show that $p_{nt} \approx q$ and $p_t \approx q\gamma$. Thus we have the approximate distributions for X_d, X_u and X_t:

$$X_d \sim \text{Pois}(2\mu\gamma N_d) \qquad X_{nt} \sim \text{Pois}(q N_d) \qquad X_t \sim \text{Pois}(q\gamma N_d). \quad (4)$$

Because an estimate of the mutation rate μ can be obtained from other sources, for each gene, we assume μ is known, but q and γ need to be estimated.

For the case/control data, the number of Aa genotypes in the cases and controls follow a binomial distribution: $X_{\text{cs}} \sim \text{Bin}(N_{\text{cs}}, p_{\text{cs}})$ and $X_{\text{cn}} \sim \text{Bin}(N_{\text{cn}}, p_{\text{cn}})$. Following the standard analysis of case/control data [1], it is easy to show that for rare variants:

$$p_{\text{cs}} = q\gamma \frac{1-K}{1-\gamma K} \approx q\gamma, \qquad p_{\text{cn}} = q, \quad (5)$$

where K is the disease prevalence. Again, assuming q is small, the binomial distribution can be approximated by the Poisson distribution:

$$X_{\text{cs}} \sim \text{Pois}(q\gamma N_{\text{cs}}), \qquad X_{\text{cn}} \sim \text{Pois}(q N_{\text{cn}}). \quad (6)$$

The similarity of the distributions of case/control and transmitted/nontransmitted data suggests a natural way to combine the two types of data. Focus on the type of mutation we are studying (e.g. LoF) and let $X_0 = X_u + X_{\mathrm{cn}}$ be the total number of LoF variants in the controls plus the number of nontransmitted LoF variants, and $X_1 = X_t + X_{\mathrm{cs}}$ be the total number of LoF variants in the cases plus the number of transmitted LoF variants, we have:

$$X_d \sim \mathrm{Pois}\,(2\mu\gamma N_d) \qquad X_1 \sim \mathrm{Pois}\,(q\gamma N_1) \qquad X_0 \sim \mathrm{Pois}(qN_0), \tag{7}$$

where $N_0 = N_{\mathrm{cn}} + N_d$ and $N_1 = N_{\mathrm{cs}} + N_d$. For any gene, the likelihood function is simply the product of the three individual probabilities:

$$P(x_d, x_1, x_0|q, \gamma) = \mathrm{Pois}(x_d|2\mu\gamma N_d) \cdot \mathrm{Pois}(x_1|q\gamma N_1) \cdot \mathrm{Pois}(x_0|qN_0). \tag{8}$$

2 The impact of mutation rates on the Multiplicity Test

Our model states that the number of *de novo* mutations per gene (X) follows Poisson distribution, $X \sim \mathrm{Pois}\,(2\mu\gamma N)$, where μ is the mutation rate of the gene (of the type of mutation we are studying, e.g. LoF), N is the number of family trios and γ is the relative risk of the mutation (1 for non-risk genes). Let $\lambda = 2\mu\gamma N$ be the Poisson rate. We introduce an approximation for Poisson distribution when the rate parameter λ is small:

$$P(X \geq 2|\lambda) = 1 - e^{-\lambda} - \lambda e^{-\lambda} \approx \lambda^2/2, \tag{9}$$

where we use the second-order Taylor expansion: $e^{-\lambda} \approx 1 - \lambda + \lambda^2/2$, which suggests that the probability of having a recurrent mutation increases quadratically with the mutation rate μ.

This observation has two consequences. First, it means that the power of detecting high-mutation rate (usually large) genes by the Multiplicity Test is much higher than for smaller genes, and it explains our observations in Figure 1A. Second, it also means that the same double-hit (two *de novo* mutations in one gene) should be assigned different meaning or p-value for genes of different sizes, as it is easier to achieve a double-hit by chance for large genes. The De Novo Poisson Test adjusts the mutation rate difference across genes. For instance, in Table S3, we see that the gene CHD8 has a relatively high mutation rate 0.0001 (the genome-wide average is about 2.4×10^{-5}), and its p-value from the De Novo Test is about 0.0001; on the other hand, KATNAL2 has below-average mutation rate, and its p-value from the De Novo Test is much smaller at 3.1×10^{-6}.

3 Bayesian inference of risk genes

In this section, we focus on data from a single type of mutation (LoF or damaging missense) and discuss how to combine the evidence from the two types of mutations later. To infer if a gene is a causal gene of the disease, we compare two hypothesis, $H_1 : \gamma \neq 1$ vs. $H_0 : \gamma = 1$. For most genes, the number of LoF mutations are generally very small (often 0), so the likelihood-based method may produce incorrect or unrealistic values for the parameters, e.g. $\hat{q} = 0$. Thus we take a Bayesian approach. Let $x = (x_d, x_1, x_0)$ be the count data for a gene to be tested, and let q and γ be the population frequency and relative risk, respectively. We model the prior of q and γ using the Gamma distribution, which is conjugate to the Poisson distribution:

$$\gamma|H_1 \sim \mathrm{Gamma}(\alpha, \beta) \qquad q|H_1 \sim \mathrm{Gamma}(\rho, \nu) \qquad q|H_0 \sim \mathrm{Gamma}(\rho_0, \nu_0). \tag{10}$$

Note that we use different priors for q under H_1 and H_0. This reflects the possibly different selection pressure on a risk gene, which would result in a different population frequency. The model evidence

$P(x|H_1)$ and $P(x|H_0)$ are given by:

$$P(x|H_0) = \int p(x|q, \gamma = 1)p(q|H_0)dq, \tag{11}$$

and

$$P(x|H_1) = \int p(x|q, \gamma)p(q|H_1)p(\gamma|H_1)dqd\gamma. \tag{12}$$

The computation of the model evidence can be simplified by taking advantage of the fact that the Gamma distribution is the conjugate prior for the Poisson distribution. First, for the model evidence of H_0, we note that $P(x_d|H_0)$ can be factored out because its distribution does not depend on q. The probability of $P(x_1, x_0|H_0)$ can be computed using the marginal distribution of the Poisson random variable (integrating the rate parameters):

$$P(x_1, x_0|H_0) = \frac{N_1^{x_1} N_0^{x_0}}{x_1! x_0!} \prod_{k=1}^{x_1+x_0} (\rho_0 + k - 1) \frac{\nu_0^{\rho_0}}{(\nu_0 + N_1 + N_0)^{\rho_0 + x_1 + x_0}}. \tag{13}$$

Multiply this with $\text{Pois}(x_d|2\mu N_d)$, we have:

$$P(x|H_0) = C(x, \mu)R(\rho_0, x_1 + x_0)e^{-2\mu N_d} \frac{\nu_0^{\rho_0}}{(\nu_0 + N_1 + N_0)^{\rho_0 + x_1 + x_0}}, \tag{14}$$

where we have the constant terms (not dependent on the parameters):

$$C(x, \mu) = \frac{N_1^{x_1} N_0^{x_0} (2\mu N_d)^{x_d}}{x_1! x_0! x_d!}, \tag{15}$$

and

$$R(\rho_0, x_1 + x_0) = \prod_{k=1}^{x_1+x_0} (\rho_0 + k - 1). \tag{16}$$

The R term is 1 if $x_1 + x_0 = 0$. For the model evidence of H_1, we use the iterated integral:

$$\begin{aligned} P(x|H_1) &= \int p(\gamma|M_1)p(x_d|\gamma) \left[\int p(x_1, x_0|q, \gamma)p(q|M_1)dq\right] d\gamma \\ &= \int \text{Gamma}(\gamma|\alpha, \beta)\text{Pois}(x_d|2\mu\gamma N_d)P(x_1, x_0|\gamma)d\gamma. \end{aligned} \tag{17}$$

Integration over q is similar to the integration under H_0 above:

$$P(x_1, x_0|\gamma) = \frac{(\gamma N_1)^{x_1} N_0^{x_0}}{x_1! x_0!} \prod_{k=1}^{x_1+x_0} (\rho + k - 1) \frac{\nu^{\rho}}{(\nu + \gamma N_1 + N_0)^{\rho + x_1 + x_0}}. \tag{18}$$

Plugging in the relevant terms, we have:

$$P(x|H_1) = \frac{\beta^\alpha}{\Gamma(\alpha)} C(x, \mu)R(\rho, x_1 + x_0)\nu^\rho \int f(\gamma)d\gamma, \tag{19}$$

where

$$f(\gamma) = \frac{\gamma^{\alpha + x_1 + x_d - 1} e^{-(\beta + 2\mu N_d)\gamma}}{(\nu + \gamma N_1 + N_0)^{\rho + x_1 + x_0}}. \tag{20}$$

The integration is performed numerically. Once we have the model evidence, the role of gene can be inferred by Bayes factor:

$$B = \frac{P(x|H_1)}{P(x|H_0)}. \tag{21}$$

In general the Bayes factor is somewhat sensitive to the values of the prior parameters, so we estimate the empirical p-value of the Bayes factor using simulations. Specifically, given the count of LoF variants of the test gene, we estimate the population frequency, q, of the mutant allele assuming that the gene is not associated with the disease. Given the prior distribution $q|H_0 \sim \text{Gamma}(\rho_0, \nu_0)$, its posterior distribution, given the counts x_1 and x_0 (x_d is not related to q), follows: $q \sim \text{Gamma}(\rho_0 + x_0 + x_1, \nu_0 + N_0 + N_1)$. Thus our point estimate of q is its posterior expectation:

$$\hat{q}|H_0 = \frac{\rho_0 + x_1 + x_0}{\nu_0 + N_1 + N_0}. \tag{22}$$

We obtain the null distribution of the Bayes factor by simulating data under the estimated q and $\gamma = 1$, according to Equation 7.

4 Estimation of the prior parameters by the Hierarchical Bayes method

The Bayes inference procedure described above depends on some prior parameters. For ease of notation, we reparameterize the prior distributions using the expectations: $\bar{\gamma} = \alpha/\beta$, $\bar{q}_1 = \rho/\nu$ and $\bar{q}_0 = \rho_0/\nu_0$. We thus have the hyperparameters to be estimated: $\phi = (\bar{\gamma}, \beta, \bar{q}_1, \nu_1)$ for H_1 and $\phi_0 = (\bar{q}_1, \nu_0)$ for H_0. We estimate the values of these parameters by joint analysis of all genes in our dataset, following a hierarchical Bayes strategy [2]. Specifically, we assume a mixture model on all genes, with each gene having probability π to be a risk gene. The marginal likelihood for all genes, as a function of ϕ_1 and ϕ_0, is thus:

$$P(\mathbf{x}|\phi_1, \phi_0) = \prod_{i=1}^{m} \left[\pi P(x_i|\phi_1) + (1-\pi) P(x_i|\phi_0)\right], \tag{23}$$

where m is the total number of genes, $\mathbf{x}$ represents the count data of all genes, and x_i the count data of the i-th gene. The model evidence $P(x_i|\phi_0)$ and $P(x_i|\phi_1)$ is given by Equation 14 and 19, respectively. We assume the value of π (the fraction of risk genes) is known and maximize the marginal likelihood to obtain the values of ϕ_1 and ϕ_0.

5 TADA for multiple types of mutations

When analyzing multiple types of mutations (LoF and Mis3 in our analysis of ASD data), we assume the data from each type of mutation are independent of each other. In the first step, we estimate the prior parameters of each type of mutation using the hierarchical Bayes method. Next, we compute the Bayes factor of any gene, as the product of the Bayes factor from each type of mutation. In the case of two types of mutations, we simply have:

$$B = B_{\text{LoF}} \cdot B_{\text{mis3}}. \tag{24}$$

In our analysis of ASD data, we note that the mis3 mutations annotated in the data are probably a mixture of those causing protein-damaging changes that increase the risk of ASD and those having no real effects on the phenotype. For example, none of the five genes with recurrent LoF have $x_1 > x_0$ for mis3 mutations (Table 1, Table S3).

We developed a mixture model for the Mis3 data. Let x be the count data of mis3 variants (*de novo* count, and the counts from the case-control and inherited data), I be an indicator variable of whether the mis3 mutations are informative or not, and D be the indicator variable for whether the gene is associated with the disease. Also let w be the mixture ratio, i.e., $P(I|D)$, the probability the mis3 data are informative given that the gene is related to the disease. The BF is then defined as:

$$B = \frac{P(x|D)}{P(x|\bar{D})} = \frac{P(x|I,D)P(I|D) + P(x|\bar{I},D)P(\bar{I}|D)}{P(x|\bar{D})} = w\frac{P(x|I,D)}{P(x|\bar{D})} + (1-w), \tag{25}$$

where $P(x|I,D)/P(x|\bar{D})$ is simply the BF defined before. So our procedure of computing the new BF for a gene is:

$$B = B_{\mathrm{LoF}} \cdot [wB_{\mathrm{Mis3}} + (1-w)], \tag{26}$$

where B_{LoF} and B_{Mis3} are the BFs computed from LoF and Mis3 data, without the benefit of mixture modeling.

6 Method of moment estimation of the number of ASD genes and their relative risks

We take advantage of our probabilistic model of *de novo* mutations to estimate the number of ASD genes. Suppose we have m genes in the genome, k of which are ASD genes (the set denoted as A), and $m-k$ are non-ASD genes (the set denoted as A^c). The sample size is $N = 932$ in this study. For the i-th gene, its rate of LoF mutation is μ_i, and its relative risk is γ_i (equal to 1 for non-ASD genes). The value of μ_i is known, and γ_i is assumed to follow the distribution: Gamma(α,β). We write: $\alpha = \bar{\gamma}\beta$, where $\bar{\gamma}$ is the average relative risk of all ASD genes. Let C be the observed number of LoF events in all genes, and M be the observed number of multi-hit genes (the genes with recurrent LoF mutations). Our goal is to estimate k and $\bar{\gamma}$ from the observed values of C and M.

Following our model, Equation 4, the expected number of LoF events in all genes is given by:

$$2N\sum_{i\in A}\gamma_i\mu_i + 2N\sum_{i\in A^c}\mu_i = C. \tag{27}$$

Assuming μ_i and γ_i are independent, we take the expectation on both sides:

$$2Nk\bar{\gamma}\bar{\mu} + 2N(m-k)\bar{\mu} = C. \tag{28}$$

This leads to the constraint relating k and $\bar{\gamma}$:

$$(\bar{\gamma}-1)k = \frac{C}{2N\bar{\mu}} - m. \tag{29}$$

To estimate the number of multi-hit genes, we define p_i as the probability of the i-th gene having more than one LoF event in the *de novo* data. If the gene is an ASD gene, this probability is:

$$p_i^{(1)} = P(X_i \geq 2|i \in A) = \int \mathrm{Pois}(X_i \geq 2|2N\gamma_i\mu_i)\mathrm{Gamma}(\gamma_i|\bar{\gamma}\beta,\beta)d\gamma_i, \tag{30}$$

where X_i is the number of *de novo* LoF events in this gene across N families. If the gene is not associated with ASD, the probability is:

$$p_i^{(0)} = P(X_i \geq 2|i \in A^c) = \mathrm{Pois}(X_i \geq 2|2N\mu_i). \tag{31}$$

Summing over all ASD and non-ASD genes:

$$\sum_{i \in A} p_i^{(1)} + \sum_{i \in A^c} p_i^{(0)} = M. \tag{32}$$

Taking the expectation in both sides, we have:

$$k\mathrm{E}\left(p|\bar{\gamma}, \beta\right) + (m-k)\mathrm{E}\left(p|\gamma = 1\right) = M, \tag{33}$$

where the two expectation terms are the average of the multi-hit probability of all genes under the ASD and non-ASD cases, respectively:

$$\mathrm{E}\left(p|\bar{\gamma}, \beta\right) = \frac{1}{m}\sum_{i=1}^{m} p_i^{(1)} \qquad \mathrm{E}\left(p|\gamma = 1\right) = \frac{1}{m}\sum_{i=1}^{m} p_i^{(0)}. \tag{34}$$

Given the values of $\bar{\gamma}$ and β, these expectation terms can be easily calculated according to Equations 30 and 31. There are three unknowns in Equations 29 and 33: k, $\bar{\gamma}$ and β, so they cannot be uniquely determined. We explore the values of k and $\bar{\gamma}$ under different choices of β. In our analysis of ASD data, we fix $\beta = 1$, and estimate k and $\bar{\gamma}$ accordingly.

7 Comparison with an earlier estimate of the number of ASD genes

In an earlier paper, it was estimated that the number of ASD genes (k) is about 370 [3]. This is considerably lower than our estimate (nearly 1,000). We first review the methodology of the earlier work and then explain that it may underestimate k by treating all genes as equal. Specifically, the earlier paper uses the same *de novo* data as ours, including 127 *de novo* LoF events. By comparing with the rate of *de novo* LoF events in the unaffected siblings, it was estimated that about half of them (65) happened in ASD genes. Among these 65 genes, 5 genes have two *de novo* LoF mutations. Let the number of ASD genes be k. The data suggests that if we sample from the k genes (with replacement) 65 times, five genes will be sampled twice. For each ASD gene, let X be the number of times it was sampled, then X follows Binomial distribution with probably $1/k$. So we have:

$$k \cdot P(X \geq 2|\mathrm{Bin}(65, 1/k)) = 5. \tag{35}$$

Solving the equation gives $k = 370$.

Clearly, in the above analysis, the probability of sampling one ASD gene is assumed to be equal. In fact, if we assume that all genes have the same mutation rates, and all ASD genes have the same relative risks, solving Equations 29 and 33 would lead to $k = 376$. We show that assuming equal mutation rates and equal relative risks significantly underestimate k. First, we use an approximation for the Poisson distribution introduced earlier (Equation 9), when the rate parameter λ is small:

$$P(X \geq 2|\lambda) = 1 - e^{-\lambda} - \lambda e^{-\lambda} \approx \lambda^2/2. \tag{36}$$

Applying this approximation to the probability of having multiple *de novo* mutations under the ASD and non-ASD scenarios (Equations 30 and 31, assuming a fixed γ_i):

$$p_i^{(1)} = 2N^2\gamma_i^2\mu_i^2 \qquad p_i^{(0)} = 2N^2\mu_i^2. \tag{37}$$

Taking the expectation of $p_i^{(1)}$ and $p_i^{(0)}$, and plug in the terms into Equation 33:

$$2kN^2 \cdot \mathrm{E}\left(\gamma^2\right)\mathrm{E}\left(\mu^2\right) + 2(m-k)N^2 \cdot \mathrm{E}\left(\mu^2\right) = M. \tag{38}$$

We can then solve for $\mathrm{E}(\gamma^2)$:

$$\mathrm{E}(\gamma^2) = \frac{M}{2kN^2\mathrm{E}(\mu^2)} - \frac{m-k}{k} \leq \frac{M}{2kN^2[\mathrm{E}(\mu)]^2} - \frac{m-k}{k}. \tag{39}$$

where the inequality above is based on the Jensen's Inequality: $[\mathrm{E}(\mu)]^2 \leq \mathrm{E}(\mu^2)$. This suggests that if we assume all genes have equal mutation rate, and replace $\mathrm{E}(\mu^2)$ above with $[\mathrm{E}(\mu)]^2$, we would overestimate $\mathrm{E}(\gamma^2)$. Again by Jensen's inequality, we know that $\mathrm{E}(\gamma)^2 \leq \mathrm{E}(\gamma^2)$. Taken together, we have:

$$\mathrm{E}(\gamma) \leq \sqrt{\mathrm{E}(\gamma^2)} \leq \sqrt{\mathrm{E}(\gamma^2|\text{assuming equal } \mu_i)}. \tag{40}$$

Thus, the actual average γ is always lower than the estimate based on the assumption that all genes have the same mutation rates and all ASD genes have the same relative risks. Because of the relationship between k and γ (Equation 29), overestimation of γ leads to an underestimation of k.

8 Parameterization in the analysis of ASD data

We first discuss how we estimate the parameters of the Hierarchical Bayes (HB) model displayed in Figure 6 for the LoF data. We use a two-step procedure.

1. Using only the *de novo* data we identify a range of values of k, denoted as R_k, that are consistent with our observations ($M = 5, C = 123$). From the method of moments analysis, by varying M from 4 to 6 we estimate that there are $R_k = [550, 1000]$ ASD genes (main text, Figure 3B). For each $k \in R_k$, the fraction of ASD genes $\pi(k) = k/18,900$ (18,900 is the number of human genes), and the corresponding $\bar{\gamma}(k)$ is determined by Equation 29. For example, at $k = 550$, we have $\bar{\gamma} = 38.5$, and at $k = 1000$, we have $\bar{\gamma} = 20.2$.

2. Using the transmitted and case-control data, we performed the HB analysis for a grid of values of $k \in R_k$ ($k = 550, 600, \cdots, 1000$) and the corresponding values of $\pi(k)$ and $\bar{\gamma}(k)$. We maximize the marginal likelihood defined by Equation 23. In the HB analysis, the other parameters in ϕ_1 and ϕ_0 are free parameters, but we limit the search range of the variance parameters of H_1: $\beta \leq 1$, and $\nu_1 \leq 10000$ (our simulations suggest that the likelihood becomes unstable outside of this range). We found that the likelihood was maximized at $k = 1000$. Setting $k = 1000$, the resulting values of the hyperparameters from the HB optimization are shown in Table S1.

We also performed a heuristic estimate of $\bar{q}_1$ (the mean LoF frequency of ASD genes) to check its consistency with the HB estimations. We analyze the list of 111 genes that have at least one *de novo* LoF mutation in the combined 932 families, and the counts of the LoF mutations of these genes in the AASC controls. It has been estimated that about half of these genes are ASD genes [3]. In the control sample we observe 64, 20 and 27 genes which have 0, 1 and ≥ 2 LoF hits, respectively. Based on the high penetrance of LoF mutations, we infer that genes with 0 counts in the controls are much more likely ASD candidates than those with 2 or more counts. The 20 single-count genes create more ambiguity. Four of these have 2 or more counts in cases, rendering them likely ASD candidates as well. This simple analysis suggests that in the 55 candidate ASD genes, somewhere between 4 and 20 LoF hits were observed, providing an interval estimate for $\bar{q}_1$ of 8.4×10^{-5} to 4.2×10^{-4}. Furthermore, we note that the genes with higher mutation rates are more likely to have *de novo* LoF mutations, thus the group of 111 genes should be enriched with high mutation-rate genes. Because we are estimating the average q_1 of all ASD genes, we need to

correct for the higher mutation rates in our list. We estimate via simulation the mean mutation rate of genes, given that the genes have at least one *de novo* LoF. It is about 1.7 times higher than average. We assume that the population frequency is proportional to μ (from mutation-selection balance in population genetics, $q = \mu/s$, where s is the selection coefficient), thus we correct $\bar{q}_1$ by dividing 1.7, and this leads to an estimate of $\bar{q}_1$ from 4.9×10^{-5} to 2.4×10^{-4} for ASD genes.

For Mis3 mutations, we first estimate w in Equation 26, the fraction of informative Mis3 mutations. We compile a small list of highly confident ASD genes. (1) We first choose the most likely ASD genes from a list of about 100 candidates from a recent review paper [4]: TSC2, TSC1, NLGN4X, SCN1A, CNTNAP2, CHD7, CACNA1C and TBX1. (2) We intersect this list with the list of genes with at least one *de novo* LoF mutation in the probands, and this results in five additional genes: FOXP1, GRIN2B, MBD5, NRXN1, FMR1. (3) We choose all five double-hit genes: KATNAL2, CHD8, DYRK1A, POGZ and SCN2A (Table 1, Table S3). (4) We add two additional genes TBR1 and CUL3 that have been replicated recently [5, 6]. In this total list of 20 genes: 11 genes have more Mis3 mutations in cases than in controls. So we estimate that the Mis3 data are informative in about 55% of the risk genes. We thus choose $w = 0.55$ in experiments.

Assuming $k = 1000$ ASD genes from the LoF analysis, we estimate the average $\bar{\gamma}$ of Mis3 mutations using Equation 29. Specifically, we observe $E = 319$ *de novo* Mis3 mutations in probands. The expected number of mutations, $2N\bar{\mu}m$, is 253.7 (number of *de novo* Mis3 mutations in siblings, normalized by the number of siblings). There is clearly, however, some uncertainty of this estimate, so we allow a 5% error of this expected number (241 to 266), and this leads to a range of $\bar{\gamma}$ in $(4.73, 7.12)$. We then search for the best $\bar{\gamma}$ in this range with the HB method. Similar to the LoF analysis, we restrict the variance parameters of γ and q under H_1: $\beta \leq 1$, $\nu_1 \leq 2000$ (ν_1 is lower to allow more variance of q, which is expected for missense mutations, as opposed to LoF mutations).

In summary, the parameters we use in the predictions are $k = 1000$ genes, or $\pi = 1000/18,900 = 0.053$, the weight of the Mis3 mutations, $w = 0.55$, and the hyperparameters for the LoF and Mis3 mutations are listed in Table S1.

9 Controlling FDR in simulations

In our simulations, we apply the three tests, De Novo, Meta and TADA, to the data simulated from the procedure described in Methods. We describe how we control FDR for each test. Specifically, for any simulated dataset (about 18,000 genes), we calculate M_1, the number of true discoveries, and FDR, under various significance levels. At any significance level α, we count the number of genes with p-value below α and denote it by M_+. We also estimate the number of false positives at the level α, M_0. For the Meta and TADA tests, this is simply:

$$M_0 = (m - k) \cdot \alpha. \tag{41}$$

where $m = 18,000$ is the total number of genes, and $k = 1,000$ is the number of risk genes. This follows from the uniform distribution of p-values under the null model. For the De Novo test, because p-values are generally discrete (a gene can have 0, 1 or 2 *de novo* LoF mutations), they do not follow the uniform distribution; however, we can easily estimate M_0 as follows. For the i-th gene, let p_i be the probability that it reaches a p-value below α, assuming it is not related to the disease. Because we know that the count of genes under the null model follows the distribution $X_i \sim \text{Pois}(2\mu_i N)$, this count is easily calculated for any α. Consequently we have:

$$M_0 = (m - k) \cdot \frac{1}{m} \sum_{i=1}^{m} p_i. \tag{42}$$

Once M_0 is estimated, we have $M_1 = M_+ - M_0$ for any significance level α, and the FDR is M_0/M_+. To meet the FDR target 0.1, we choose the largest α that leads to FDR below 0.1, and obtain the corresponding M_1 as the number of true discoveries.

10 Genomic control for TADA

In Liu [7] it was noted that a variation on the traditional genomic control (GC) factor performs better when the test statistic follows the assumed distribution for small p-values, but does not closely follow this distribution for p-values that are not of interest. TADA offers an example of this phenomenon: for a substantial number of genes all LoF counts are equal to 0 creating a mass of statistics with equal p-value. (For these data, this mass occurs near .3.) Naturally, these genes are not of interest, but this mass of p-values can cause a substantial inflation in λ, as traditionally calculated.

To compute the traditional GC factor, we define p_{med} to be the median p-value and let $\lambda = F^{-1}(1 - p_{\mathrm{med}})/.455$, where F^{-1} is the inverse CDF of the χ^2_1 distribution. To obtain a more robust GC factor, let p_q be the first quantile of the p-values, and define $\lambda_q = F^{-1}(1 - p_q)/1.32$. This statistic has expectation 1 if the statistic follows the assumed model. Values bigger than 1 arise due to deviations from the assumed distribution such as would arise due to population stratification.

11 Variant Calling

De novo events were called as described in the relevant publications [8, 9, 10, 11]. Variant calling for the case-control data is described in Liu et al. [7]. To call transmitted variants for the portion of the data derived from the Simon's trios the following procedures were utilized. Whole-exome data was obtained using the NimbleGen EZ SeqCap v2 and Illumina HiSeq for 656 ASD families (473 quartets including an unaffected sibling, and 183 trios with only an affected proband). The reads were aligned to hg19 using BWA and variants were predicted using Samtools after removal of duplicate reads for all family members. An in-house script was used to exclude variants that were over 37bp off-target (i.e. 50% of the 75bp read-length from the target). Fifteen families were excluded on the basis of at least one family member having a number of predicted variants that was > 3 standard deviations from the mean leaving 641 families (1,282 parents; 460 quartets, 181 trios). All predicted substitution variants were cross-referenced with the proband and sibling data to identify the percentage of unique reads in the offspring that support the parental variant; a variant was considered transmitted if this value was greater than 20%. Parental variants were annotated against UCSC gene definitions and the Exome Sequencing Project (ESP) variant database for 6,500 control individuals [12].

To ensure high quality data the variant were filtered to include only the variants with: $\geq$ 20 unique reads in all family members; ≥ 6 unique reads supporting the variant in the parent; heterozygous variants in the parent; and a Samtools SNP quality score ≥ 50 in the parent. Common variants were excluded by removing any variant that was present at $> 1\%$ population frequency in the ESP controls and/or the 1,282 parents. After filtering, 364,445 variants remained giving a mean of 284 variants per parent.

References

[1] Nan M Laird and Christoph Lange. *The fundamentals of modern statistical genetics.* Springer, 2010.

[2] Bradley Efron. *Large-Scale Inference: Empirical Bayes Methods for Estimation, Testing, and Prediction.* Cambridge University Press, 2010.

[3] I. Iossifov, M. Ronemus, D. Levy, Z. Wang, I. Hakker, J. Rosenbaum, B. Yamrom, Y. H. Lee, G. Narzisi, A. Leotta, J. Kendall, E. Grabowska, B. Ma, S. Marks, L. Rodgers, A. Stepansky, J. Troge, P. Andrews, M. Bekritsky, K. Pradhan, E. Ghiban, M. Kramer, J. Parla, R. Demeter, L. L. Fulton, R. S. Fulton, V. J. Magrini, K. Ye, J. C. Darnell, R. B. Darnell, E. R. Mardis, R. K. Wilson, M. C. Schatz, W. R. McCombie, and M. Wigler. De novo gene disruptions in children on the autistic spectrum. *Neuron*, 74(2):285–299, Apr 2012.

[4] C. Betancur. Etiological heterogeneity in autism spectrum disorders: more than 100 genetic and genomic disorders and still counting. *Brain Res.*, 1380:42–77, Mar 2011.

[5] Brian J O'Roak, Laura Vives, Wenqing Fu, Jarrett D Egertson, Ian B Stanaway, Ian G Phelps, Gemma Carvill, Akash Kumar, Choli Lee, Katy Ankenman, Jeff Munson, Joseph B Hiatt, Emily H Turner, Roie Levy, Diana R O'Day, Niklas Krumm, Bradley P Coe, Beth K Martin, Elhanan Borenstein, Deborah A Nickerson, Heather C Mefford, Dan Doherty, Joshua M Akey, Raphael Bernier, Evan E Eichler, and Jay Shendure. Multiplex targeted sequencing identifies recurrently mutated genes in autism spectrum disorders. *Science*, Nov 2012.

[6] A. Kong, M. L. Frigge, G. Masson, S. Besenbacher, P. Sulem, G. Magnusson, S. A. Gudjonsson, A. Sigurdsson, A. Jonasdottir, A. Jonasdottir, W. S. Wong, G. Sigurdsson, G. B. Walters, S. Steinberg, H. Helgason, G. Thorleifsson, D. F. Gudbjartsson, A. Helgason, O. T. Magnusson, U. Thorsteinsdottir, and K. Stefansson. Rate of de novo mutations and the importance of father's age to disease risk. *Nature*, 488(7412):471–475, Aug 2012.

[7] L. Liu, A. Sabo, B. M. Neale, U. Nagaswamy, C. Stevens, E. Lim, C. A. Bodea, D. Muzny, J. G. Reid, E. Banks, H. Coon, M. Depristo, H. Dinh, T. Fennel, J. Flannick, S. Gabriel, K. Garimella, S. Gross, A. Hawes, L. Lewis, V. Makarov, J. Maguire, I. Newsham, R. Poplin, S. Ripke, K. Shakir, K. E. Samocha, Y. Wu, E. Boerwinkle, J. D. Buxbaum, E. H. Cook, B. Devlin, G. D. Schellenberg, J. S. Sutcliffe, M. J. Daly, R. A. Gibbs, and K. Roeder. Analysis of Rare, Exonic Variation amongst Subjects with Autism Spectrum Disorders and Population Controls. *PLoS Genet.*, 9(4):e1003443, Apr 2013.

[8] B. M. Neale, Y. Kou, L. Liu, A. Ma'ayan, K. E. Samocha, A. Sabo, C. F. Lin, C. Stevens, L. S. Wang, V. Makarov, P. Polak, S. Yoon, J. Maguire, E. L. Crawford, N. G. Campbell, E. T. Geller, O. Valladares, C. Schafer, H. Liu, T. Zhao, G. Cai, J. Lihm, R. Dannenfelser, O. Jabado, Z. Peralta, U. Nagaswamy, D. Muzny, J. G. Reid, I. Newsham, Y. Wu, L. Lewis, Y. Han, B. F. Voight, E. Lim, E. Rossin, A. Kirby, J. Flannick, M. Fromer, K. Shakir, T. Fennell, K. Garimella, E. Banks, R. Poplin, S. Gabriel, M. DePristo, J. R. Wimbish, B. E. Boone, S. E. Levy, C. Betancur, S. Sunyaev, E. Boerwinkle, J. D. Buxbaum, E. H. Cook, B. Devlin, R. A. Gibbs, K. Roeder, G. D. Schellenberg, J. S. Sutcliffe, and M. J. Daly. Patterns and rates of exonic de novo mutations in autism spectrum disorders. *Nature*, 485(7397):242–245, May 2012.

[9] B. J. O'Roak, L. Vives, S. Girirajan, E. Karakoc, N. Krumm, B. P. Coe, R. Levy, A. Ko, C. Lee, J. D. Smith, E. H. Turner, I. B. Stanaway, B. Vernot, M. Malig, C. Baker, B. Reilly, J. M. Akey, E. Borenstein, M. J. Rieder, D. A. Nickerson, R. Bernier, J. Shendure, and E. E. Eichler. Sporadic autism exomes reveal a highly interconnected protein network of de novo mutations. *Nature*, 485(7397):246–250, May 2012.

[10] S. J. Sanders, M. T. Murtha, A. R. Gupta, J. D. Murdoch, M. J. Raubeson, A. J. Willsey, A. G. Ercan-Sencicek, N. M. DiLullo, N. N. Parikshak, J. L. Stein, M. F. Walker, G. T. Ober, N. A. Teran, Y. Song, P. El-Fishawy, R. C. Murtha, M. Choi, J. D. Overton, R. D. Bjornson, N. J. Carriero, K. A. Meyer, K. Bilguvar, S. M. Mane, N. Sestan, R. P. Lifton, M. Gunel, K. Roeder, D. H. Geschwind, B. Devlin, and M. W. State. De novo mutations revealed by whole-exome sequencing are strongly associated with autism. *Nature*, 485(7397):237–241, May 2012.

[11] I. Iossifov, M. Ronemus, D. Levy, Z. Wang, I. Hakker, J. Rosenbaum, B. Yamrom, Y. H. Lee, G. Narzisi, A. Leotta, J. Kendall, E. Grabowska, B. Ma, S. Marks, L. Rodgers, A. Stepansky, J. Troge, P. Andrews, M. Bekritsky, K. Pradhan, E. Ghiban, M. Kramer, J. Parla, R. Demeter, L. L. Fulton, R. S. Fulton, V. J. Magrini, K. Ye, J. C. Darnell, R. B. Darnell, E. R. Mardis, R. K. Wilson, M. C. Schatz, W. R. McCombie, and M. Wigler. De novo gene disruptions in children on the autistic spectrum. *Neuron*, 74(2):285–299, Apr 2012.

[12] Exome Variant Server, NHLBI GO Exome Sequencing Project (ESP), Seattle, WA. http://evs.gs.washington.edu/EVS/, 2012.
